# A global atmospheric methane record from a tropical ice core

**DOI:** 10.1038/s41586-026-10938-1

**Published:** 2026-08-19

**Authors:** Kara A. Lamantia, Lonnie G. Thompson, Mary E. Davis, Ben Riddell-Young, Ivo Strawson, Emilie Beaudon, Ellen Mosley-Thompson, Newton Nguyen, Edward J. Brook

**Affiliations:** 1 Byrd Polar and Climate Research Center, Ohio State University, Columbus, OH USA; 2 School of Earth Sciences, Ohio State University, Columbus, OH USA; 3 School of Geographical Sciences, University of Bristol, Bristol, UK; 4 Cooperative Institute for Research in Environmental Sciences (CIRES), University of Colorado Boulder, Boulder, CO USA; 5 College of Earth, Ocean, and Atmospheric Sciences (CEOAS), Oregon State University, Corvallis, OR USA; 6 Doerr School of Sustainability, Stanford University, Stanford, CA USA

**Keywords:** Palaeoclimate, Climate and Earth system modelling, Cryospheric science

## Abstract

Tropical wetlands are widely considered the largest natural source of atmospheric methane (CH_4_)^1–4^. However, uncertainties about wetland extent and CH_4_ production have led to large variations in modelled CH_4_ emission trends^2,3^. Most historical reconstructions rely on data from polar ice cores, which cannot fully resolve the tropical contribution^5,6^ to the CH_4_ budget. Here we present a 2,000-year record of atmospheric CH_4_ concentrations from ice cores drilled from the South Peak summit of Nevado Huascarán (Summit Core A, SCA; −9.122° S, −77.605° W; 6,768 m asl). We find that the trends and magnitudes of our CH_4_ record are broadly consistent with polar records^7^. Our *δ*^13^C-CH_4_ measurements (from approximately 1530 CE to 1999 CE) align with isotope values^8^ consistent with a dominant tropical CH_4_ source. Integration of our record into an atmospheric four-box model suggests a sustained equatorial dominance of CH_4_ source strength over the past two millennia. Our findings indicate that equatorial CH_4_ emissions are higher than previous estimates based only on polar ice core data, supporting the long-standing hypothesis that low-latitude CH_4_ emissions dominated pre-industrial (PI) CH_4_ variability^5,6^. These results demonstrate the importance of tropical ice cores on the reconstruction of CH_4_ variability and latitudinal distribution.

## Abstract (web summary)

A 2,000-year Peruvian ice core record of atmospheric CH_4_ concentrations contains the first historical global CH_4_ record from the low latitudes, and implementation of a four-box model indicates higher equatorial CH_4_ strength than previous estimates from polar-only records.

## Main

Atmospheric CH_4_ concentrations varied between approximately 350 and 800 parts per billion (ppb) over the past 800,000 years but since 1750 CE have risen approximately 160%, reaching 1,935 ppb in 2025^9^. Estimations of past fluxes and emissions still contain uncertainty, highlighting the need for improved constraints on the role of the tropics in CH_4_ production^5,10–15^. To better understand past changes in global levels of CH_4_, the inter-polar difference (IPD) was reconstructed from the Greenland Ice Sheet Project 2 (GISP2; Greenland) and West Antarctic Ice Sheet (WAIS; Antarctica) cores^7^. From 800 to 1750 CE, the IPD is on average 44 ± 7 ppb, with GISP2 above the WAIS record^7^. This is interpreted as a reflection of the long-term dominance of Northern Hemisphere emissions. However, these polar-derived mixing ratios only indirectly reflect concentrations in the tropics, creating uncertainty as to the role of tropical sources in historical CH_4_ variability^5,6^.

Consistent, long-term CH_4_ records from low-latitude ice cores would greatly improve our understanding of the atmospheric CH_4_ history, but the few existing records are affected by notable complications. For example, a core from the Sajama ice cap in Bolivia (18° S) records large (100–900 ppb) spikes in CH_4_ concentrations that are probably linked to elevated dust levels^16^, which is an issue also documented at smaller magnitudes^17^ in Greenland cores. Methane records from the Himalayan Dasuopu glacier (28° N) show an average concentration roughly 120 ppb higher than average Greenland levels; however, frequent melt layers are thought to impose unrealistically large variability as well as high uncertainty (±37 ppb) on the CH_4_ record^18^. The Mount Everest East Rongbuk Glacier ice core (28° N)^19^ shows late PI Holocene CH_4_ that are about 36 ± 17 ppb higher than in Greenland cores, but only 15 of the original 112 values are considered valid after outlier elimination.

**Extended Data Fig. 1:**
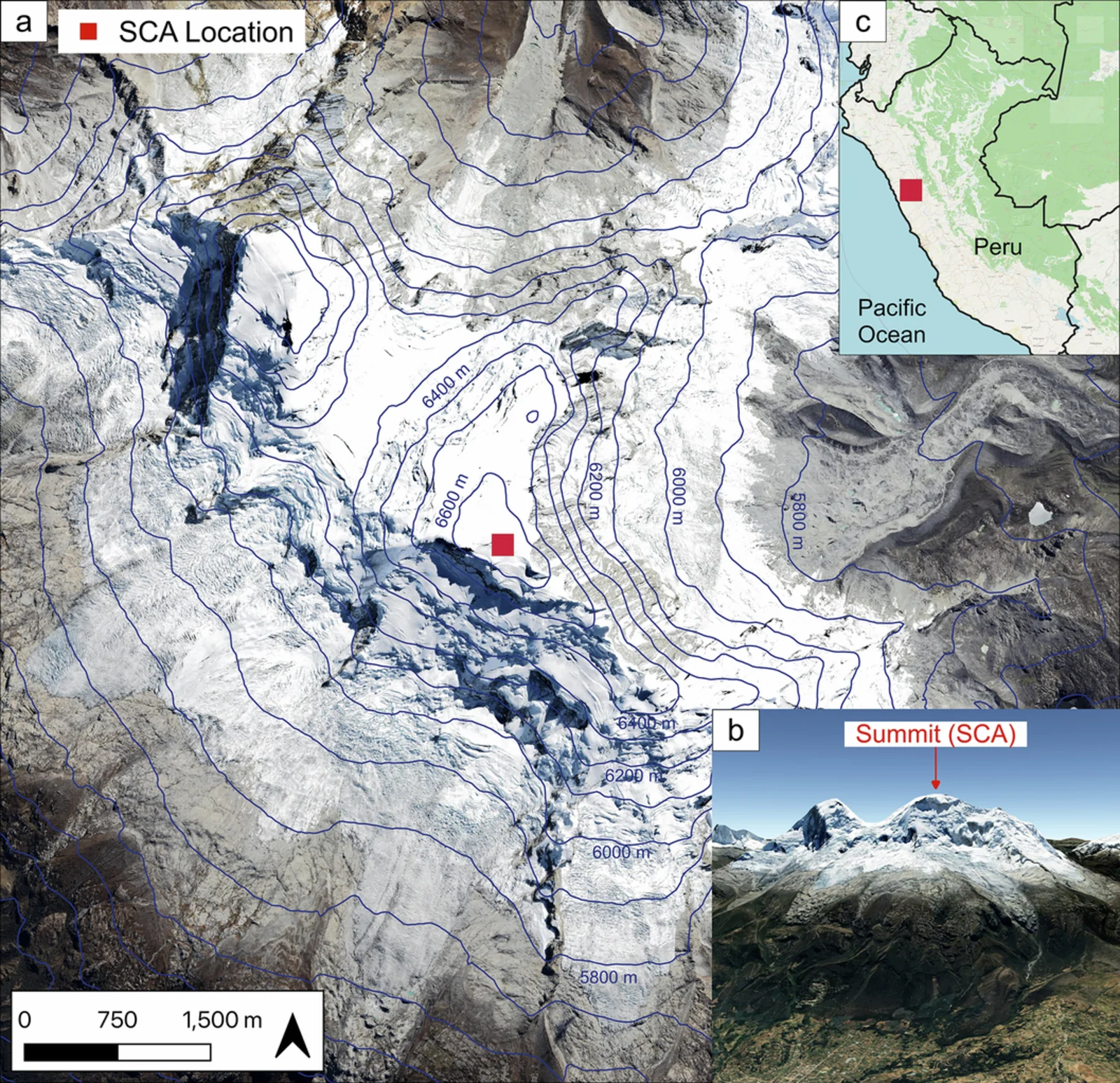
Huascarán SCA site location. **a**–**c**, Overview maps of the South Peak summit (6,768 m asl) of Nevado Huascarán (−9.122° S, −77.605° W) from aerial (**a**), vertical (**b**) and regional (**c**) perspectives showing its location on Nevado Huascarán and within Peru.

In 2019, the Byrd Polar and Climate Research Center (BPCRC) ice core research team conducted an ice core drilling programme on Nevado Huascarán (−9.122° S, −77.605° W), the world’s highest tropical mountain located in the Cordillera Blanca in the Peruvian Andes (Extended Data Fig. 1). Two cores were drilled to bedrock on the summit of the South Peak (6,768 m asl). Methane measurements were made on discrete samples from SCA, yielding the first such CH_4_ concentration record from the tropics.

## The Huascarán CH_4_ record

**Fig. 1:**
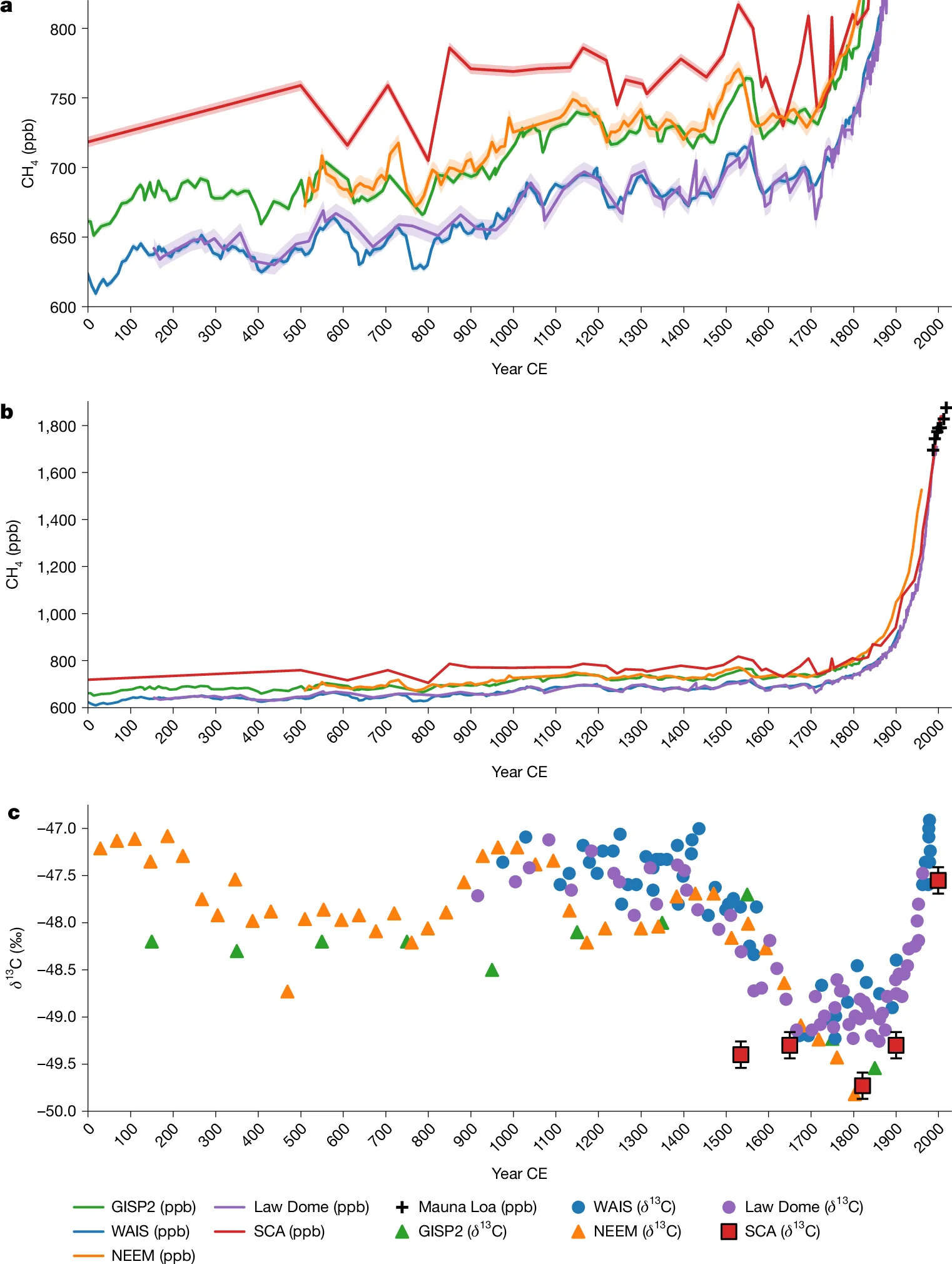
SCA and polar ice core CH_4_ concentrations and *δ*^13^C-CH_4_. **a**, Huascarán SCA CH_4_ (*n* = 51) concentrations from 0 CE to the present plotted alongside the GISP2 and WAIS records used to calculate the IPD^7^. The *y*-axis is truncated to emphasize the PI trends. NEEM^20^ and Law Dome^21^ cores are shown as 10-year averages; shading represents ±1 standard deviation (GISP2 and WAIS: ±2.4 ppb; NEEM: ±6.2 ppb; Law Dome: ±7 ppb; and SCA: ±3.7 ppb). **b**, The same dataset showing the full *y*-axis to emphasize the industrial increase and with Mauna Loa 5-year averages (black ‘+’ signs). **c**, SCA *δ*^13^C-CH_4_ data (red squares) compared with those from Greenland (triangles) and Antarctica (circles) including GISP2^33^, NEEM^32^, Law Dome^23^ and WAIS^22^ ice cores. Source data

The most recent 2,000 years of the SCA CH_4_ data are compared with CH_4_ data from the GISP2 and WAIS Divide cores^7^ to evaluate the latitudinal methane variability (Fig. 1a,b). During the PI period (0 CE to 1850 CE), the GISP2 CH_4_ record shows an average concentration that is 48 ppb higher than the WAIS Divide record, indicating dominance of Northern Hemisphere CH_4_ emissions^7^. Other polar records show comparable PI values, for example, the North Greenland Eemian Ice Drilling (NEEM) core^20^ records a PI average only 2 ppb higher than that from the GISP2 core, whereas the average from the Law Dome Antarctic record^21^ is 16 ppb higher than that from the WAIS core. The increase in CH_4_ after the PI period is the most prominent feature of the CH_4_ records over the past 2,000 years (refs. ^9,20^).

**Extended Data Fig. 2:**
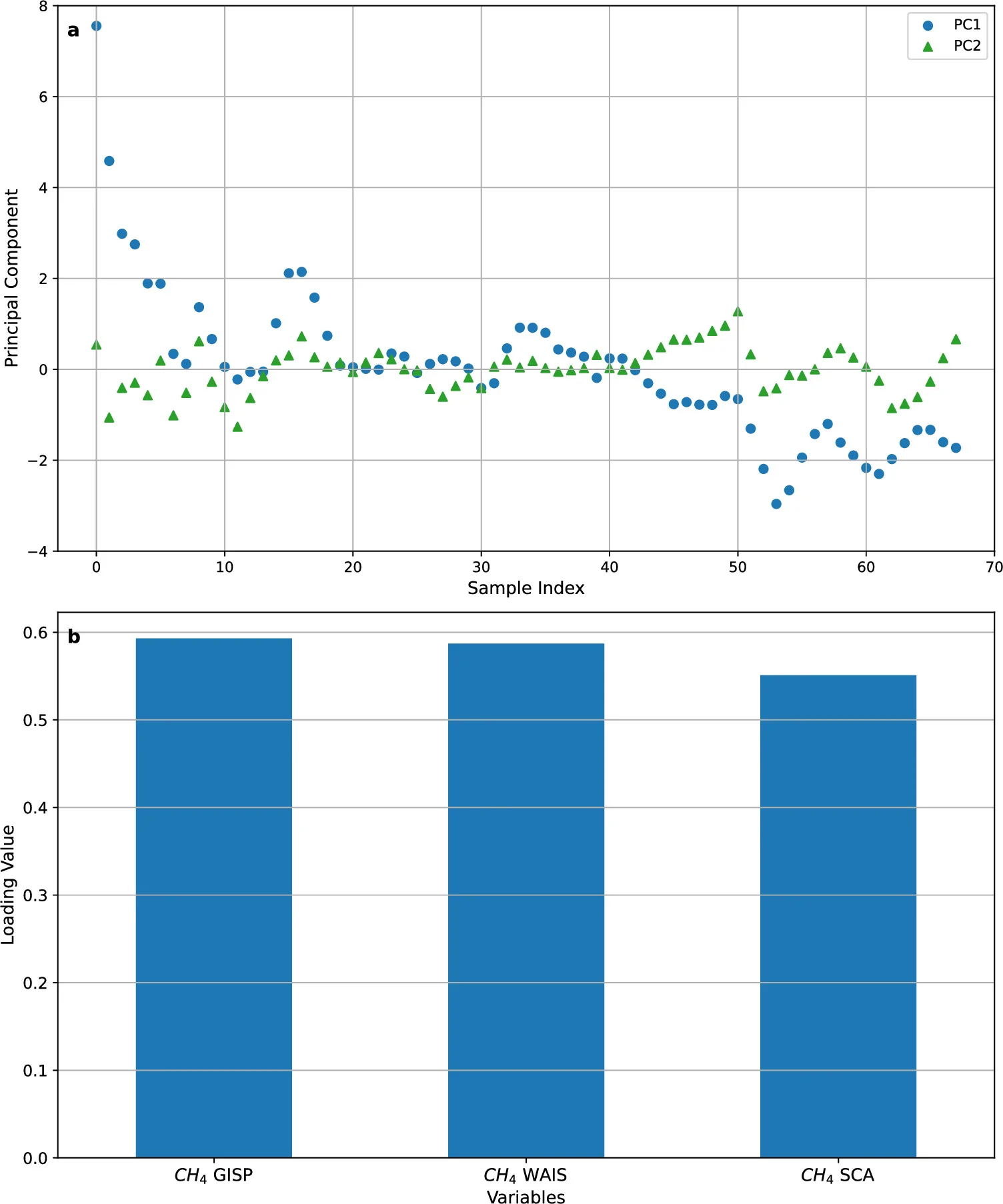
Principal component analysis. **a**, Principal component analysis results for the SCA, GISP2 and WAIS cores during the PI period with a 91% variance of the first principal component. ‘Sample index’ refers to the sample number (n = 67), starting with the most recently dated sample and extending back in time. **b**, Results of the first principal component indicating similar loading coefficients (0.59, 0.58 and 0.55, respectively). Source data

The higher methane concentrations in SCA compared with those in the polar records are expected due to the equatorial location of Huascarán and its proximity to dominant tropical sources^2,3^. The average SCA CH_4_ concentration during the PI is 46 ppb higher than the average GISP2 concentration, 48 ppb higher than NEEM, 78 higher than Law Dome and 94 ppb higher than WAIS Divide (Fig. 1a). The CH_4_ concentrations in SCA and the polar records increase concomitantly after around 1850 CE; however, the SCA values remain above those in the Law Dome core but shift approximately 30 ppb below the NEEM record (Fig. 1b). Although timescales and sampling resolutions vary, the trends and magnitudes of the SCA CH_4_ record over the past 2,000 years are broadly consistent with the polar records. One common feature in both the SCA and polar CH_4_ records is an approximately 30 ppb increase in the late fifteenth century, followed by a decrease into the seventeenth century^7,20–22^ (Fig. 1a). These types of CH_4_ variation may align with periods of historical transition, including the global increase in agricultural emissions^23,24^, the rise of the Inca farming civilization in the mid-fifteenth century and the subsequent Spanish conquest in the mid-sixteenth century^25^. Furthermore, polar records show notable decreases in CH_4_, CO_2_ and N_2_O concentrations after 1550 CE, with the minima occurring around 1600 CE (refs. ^26,27^). The SCA record shows a decrease between 1530 and 1630 CE, with CH_4_ levels declining from 817 to 730 ppb. This decrease aligns with the colloquially termed ‘Little Ice Age’^28^. However, these historical events are not necessarily the direct cause of the observed CH_4_ fluctuations but are instead helpful markers to assess existing records. Notably, the CH_4_ concentrations in the SCA, WAIS^7^ and GISP2^7^ records over the past 2,000 years exhibit high covariance of the first principal component (91%) as well as equivalent loading coefficients (Extended Data Fig. 2), suggesting that they are all influenced by the same factors that cause global CH_4_ fluctuations. A comparison between the SCA record and CH_4_ concentrations (1985–2012) from the Mauna Loa, Hawaii station^29^ indicates a ±4 ppb difference during their overlapping time periods, a further indication that the SCA record reflects global fluctuations.

Measurements of *δ*^13^C-CH_4_ (Fig. 1c) provide information about the primary sources of CH_4_, as these sources have distinct isotopic fingerprints; biogenic (for example, microbial): −50 to −70‰; pyrogenic (for example, biomass burning): −23‰; and thermogenic (for example, geologic and fossil fuels): −38 to −44‰. The combined source isotopic signature and a sink-driven kinetic isotope fractionation of about 6‰ (ref. ^30^) result in a PI global atmospheric *δ*^13^C-CH_4_ value of around −48‰ (ref. ^28^). The SCA *δ*^13^C-CH_4_ record broadly mirrors existing polar records, which show gradual depletion through the 1800s followed by a sharp enrichment after 1850 CE that is commonly linked to increasing fossil fuel emissions^8^. This trend supports the premise that SCA captures a global signal. However, the offset in the SCA isotopic values varies through time and differs between polar core records. Between approximately 1650 and 1820 CE, SCA *δ*^13^C-CH_4_ values are more isotopically depleted than those from both Greenland and Antarctic records (approximately 0.24‰ and 0.52‰, respectively). Around 1900 CE, SCA values are 0.73‰ lower than Antarctic values and lower than both Antarctic and Greenland values around 1530 CE (roughly 1.51‰)^22,23,31–33^. After accounting for the potential influence of firn fractionation^34^ (Methods), we interpret the SCA *δ*^13^C-CH_4_ values to be indicators of potentially stronger proportions of highly depleted microbial sources during the PI in the tropical regions. However, the small number of *δ*^13^C-CH_4_ samples creates some limitations to this interpretation. Sampling for this analysis was restricted because of the high demand of SCA ice for other analyses, along with the large amount of ice needed for the isotope measurements owing to low air content. Should ice be available after the conclusion of all analyses on the Huascarán summit cores, further *δ*^13^C-CH_4_ sampling would probably improve and solidify this interpretation.

**Extended Data Fig. 3:**
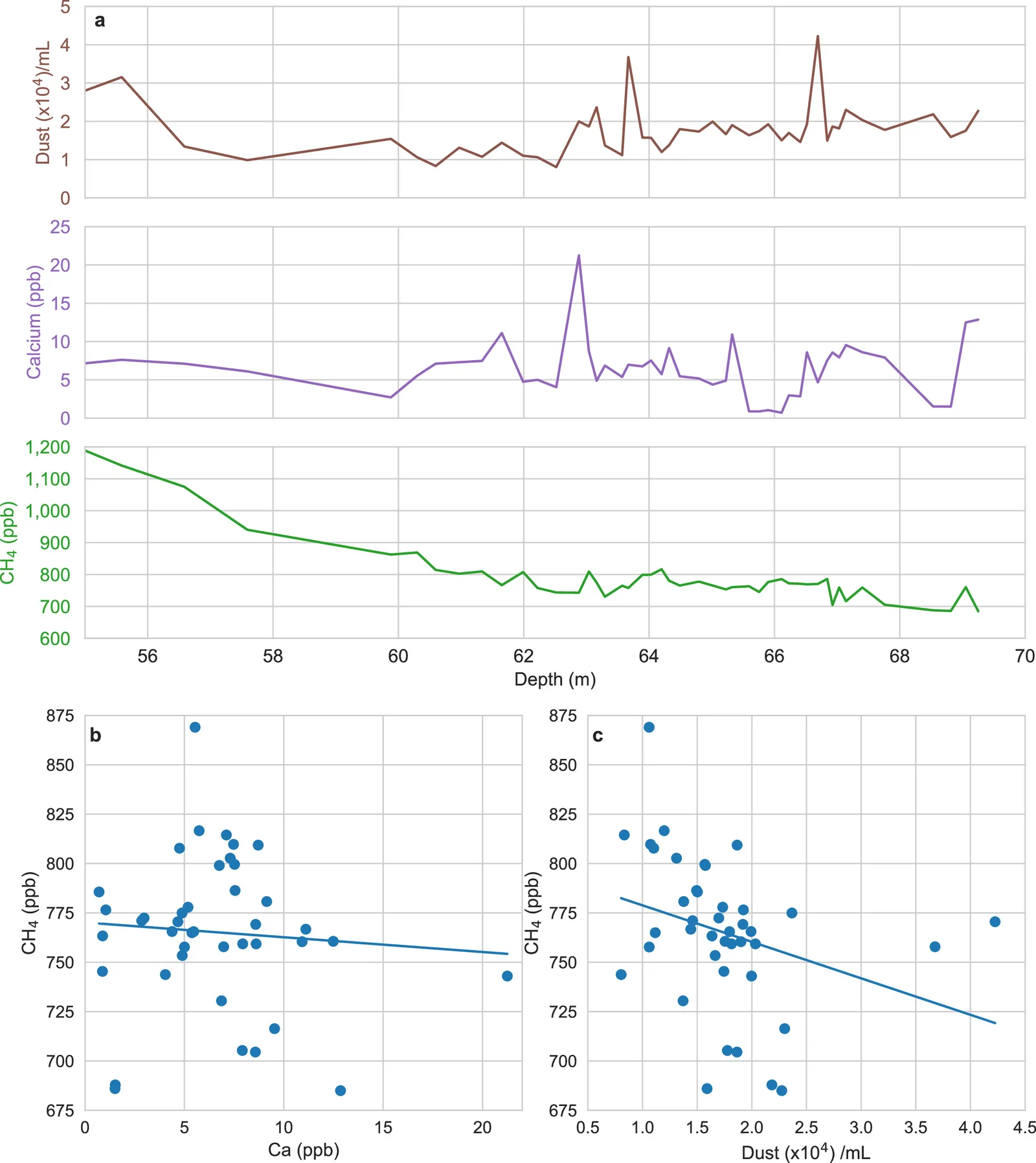
CH_4_, dust and calcium comparisons. **a**, PI SCA CH_4,_ calcium concentration and mineral dust concentration data plotted by depth (n = 45) indicating low levels of both dust and calcium and no visual similarities. **b**, A Pearson correlation with a regression line between SCA with calcium and CH_4_ levels by depth (*r* = −0.08, *P* < 0.05). **c**, A Pearson correlation with a regression line between SCA CH_4_ and dust concentrations by depth (*r* = −0.31, *P* < 0.05). Source data

**Extended Data Fig. 4:**
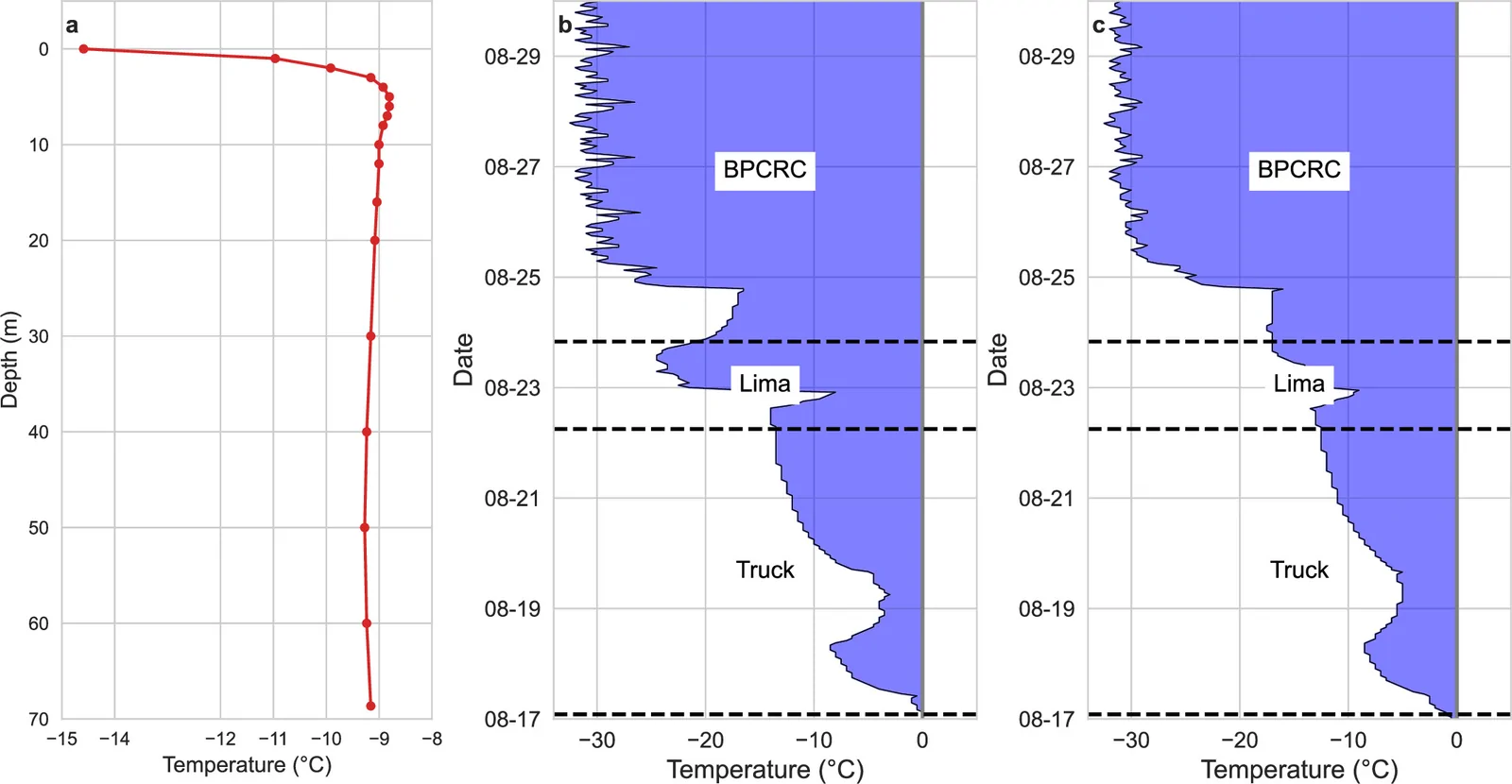
Borehole and transport temperatures. **a**, SCA borehole temperatures from surface to bedrock (n = 18) . **b**, Tube 21 thermistor hourly record. **c**, Tube 67 thermistor hourly record. The temperatures cover the period post-drilling, which involve the removal of the core tubes from their storage on Huascarán to their receipt at the BPCRC freezers. Source data

**Extended Data Fig. 5:**
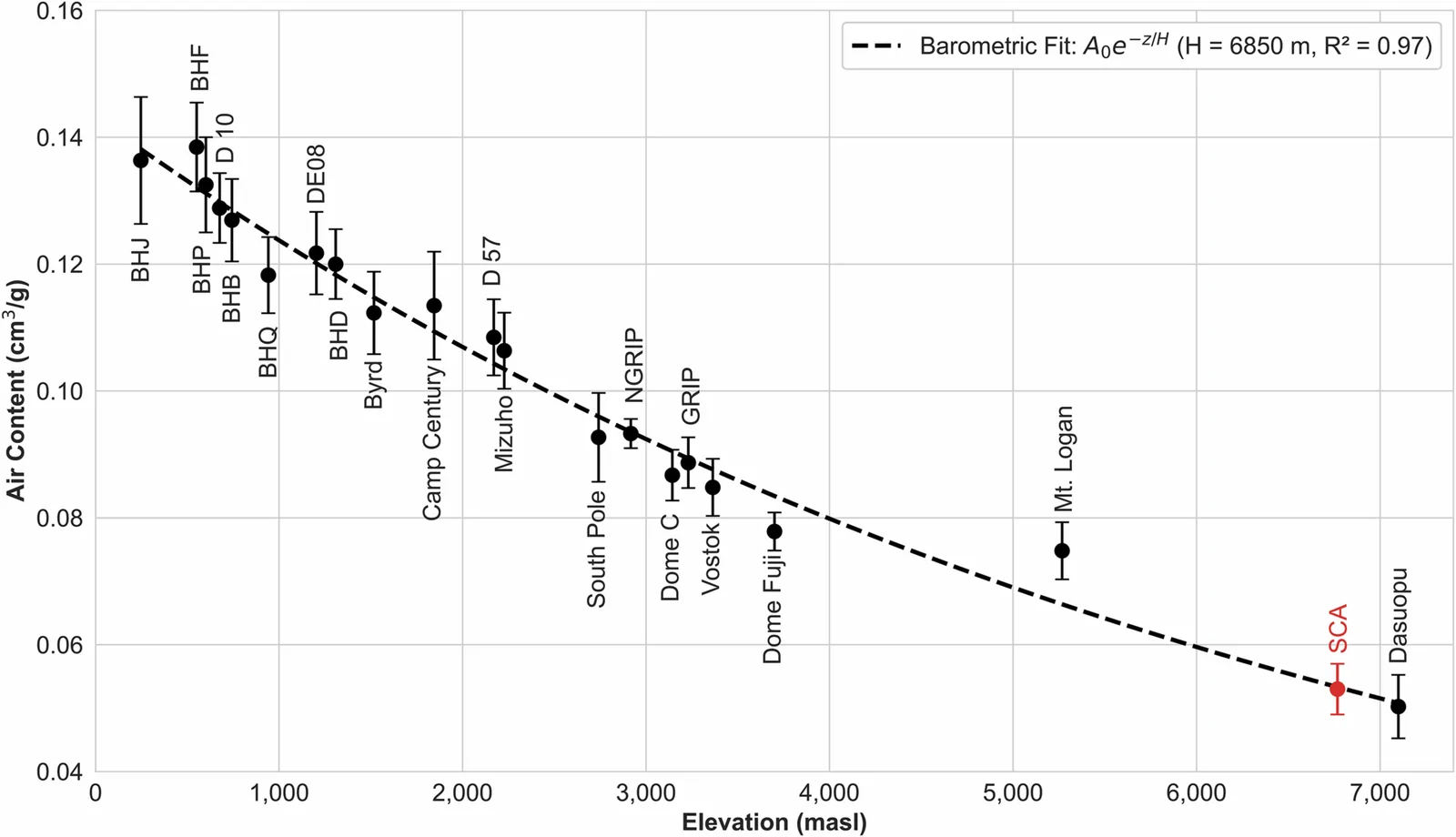
Ice core air content with barometric equation. SCA air content plotted with a barometric equation fit recording air content versus elevation in high-latitude ice cores and the Dasuopu ice core (28° N) from the Himalayas. The dots show the mean values for each drill site, with the error bars representing maximum uncertainty. From the figure, site locations for cores include the following: BHF, BHJ, BHP, BHB, BHQ and D-10 were drilled from low elevation on Law Dome, Antarctica; BHD and DE08 were drilled from the central region of Law Dome, Antarctica; D 57 is from the D 57 station in Terre Adélie, Antarctica; Byrd is from the Byrd Station in Antarctica; Mizuho is located at the Mizuho station in East Antarctica; South Pole was drilled at the South Pole Station ‘Dark Sector’; Dome C was drilled on Little Dome C in East Antarctica; NGRIP was drilled just northwest of the Greenland summit and GRIP was drilled very near to the Greenland summit; Vostok was drilled in East Antarctica; Dome Fuji was drilled in east Dronning Maud Land in Antarctica; Camp Century was drilled from northwest Greenland; Mt. Logan was drilled on Mt. Logan in Yukon, Canada; and Dasuopu was drilled on the Dasuopu glacier on the Southern Tibetan Plateau. Source data

In Greenland ice cores, excess CH_4_ is associated with dust and melt features that affect rates of pore close off that indicate melting and refreezing in the ice column^17,35^. A likely mechanism for excess CH_4_ in Greenland ice is the abiotic decomposition of organics during wet extraction analysis^36^. However, there is little evidence that any of these complications affect the SCA CH_4_ values. There is no significant correlation between SCA CH_4_ and Ca^2+^ and, although there is a moderate but negative Pearson correlation coefficient (−0.31; *P* value < 0.05) between SCA CH_4_ and dust concentration, these three records show no visual similarities to each other (Extended Data Fig. 3). The dust and Ca^2+^ concentrations in SCA are also orders of magnitude lower than in other ice cores in which dust is probably responsible for excess CH_4_ production (for example, Sajama in Bolivia^16^ and the North Greenland Ice Core Project (NGRIP) Greenland core^17^); however, the presence of dust does not have an effect on CH_4_ in Antarctic cores^27,37^. There is no evidence that temperature and air content in the Huascarán summit ice affects the SCA CH_4_ concentrations and there are no indications of visible melt layers. Borehole temperatures recorded during drilling were between −9 and −14 °C and the ice was kept frozen during transport from the drill site to its final destination at the BPCRC freezers (Extended Data Fig. 4). The air content of the SCA ice is as expected on the basis of the elevation of the drilling site and that of cores from both polar and lower latitudes^38–42^ (Extended Data Fig. 5). The correspondence between SCA and polar ice core CH_4_ trends over the past 2,000 years and the lack of evidence of CH_4_ alteration establishes SCA as the first CH_4_ low-latitude ice core to capture a consistent global CH_4_ record, whereas its slightly elevated average over polar core gas concentrations points towards the inclusion of a low-latitude tropical source.

## Constraining historical CH_4_ distribution

**Fig. 2:**
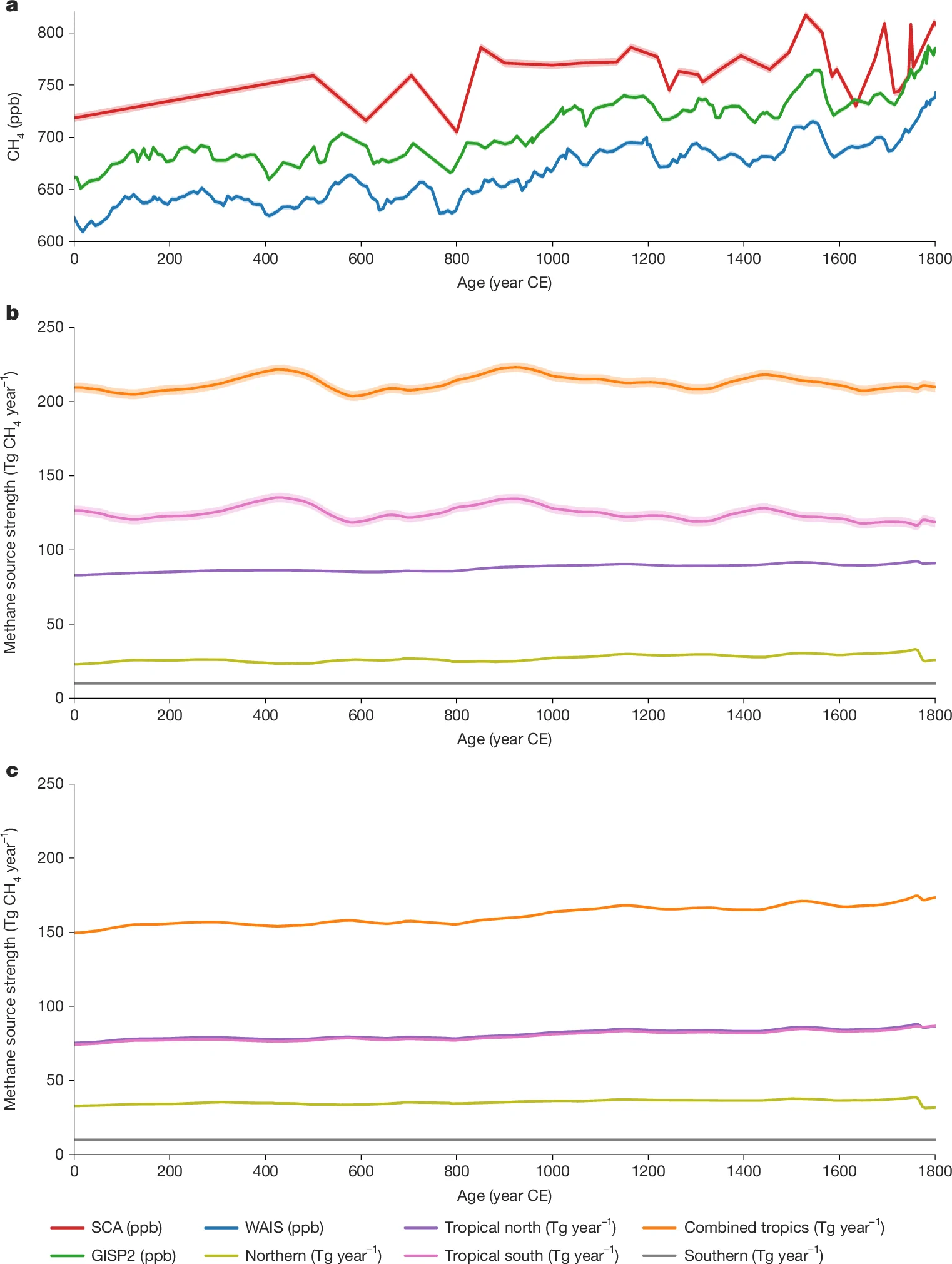
Modelled CH_4_ source strength with and without SCA data. **a**, SCA and polar ice core CH_4_ concentration data. **b**, Results from the four-box model with the addition of the SCA CH_4_ core data. **c**, Results from the four-box model using only polar records to determine CH_4_ source strength from 0 to 1800 CE. The tropical north and south boxes are summed to show a combined ‘tropics’ box for a visual indication of CH_4_ source strength in the low latitudes. Semi-transparent shading represents ±1 standard deviation from the CH_4_ (ppb) measurements (**a**) and Monte Carlo model output (**b**,**c**). Source data

To better constrain the PI CH_4_ latitudinal source strength distribution, we applied a four-box atmospheric model with the intention of partitioning sources across each of the four latitude bands. In previous implementations, the dominance of tropical source strengths remained implicitly inferred through hemispheric transport assumptions, whereas knowing from where the CH_4_ originated remained unclear^2,5,8^. With the input of only polar cores into the four-box model (Fig. 2c), the average interpolated source strength from 0 to 1800 CE for each of the tropical boxes (0–30° N and 0–30° S) are approximately 82 and 81 Tg year^−1^, respectively, and the source strength for the northern box (30–90° N) is about 36 Tg year^−1^. The addition of SCA CH_4_ into the tropical south (0–30° S) box results in a 24% increase in combined tropics source strength, from approximately 163 Tg year^−1^ to 213 Tg year^−1^ (Fig. 2b). The overall average of the tropical north box increases from 82 to 88 Tg year^−1^. We note that, in the southern box (30–90° S), the model source is fixed at a constant low level, as very few sources are noted in these latitudes^2,5^. Although the model run ends at 1800 CE, we predict that anthropogenic production of CH_4_ would create increases in the northern and tropical boxes.

**Extended Data Fig. 6:**
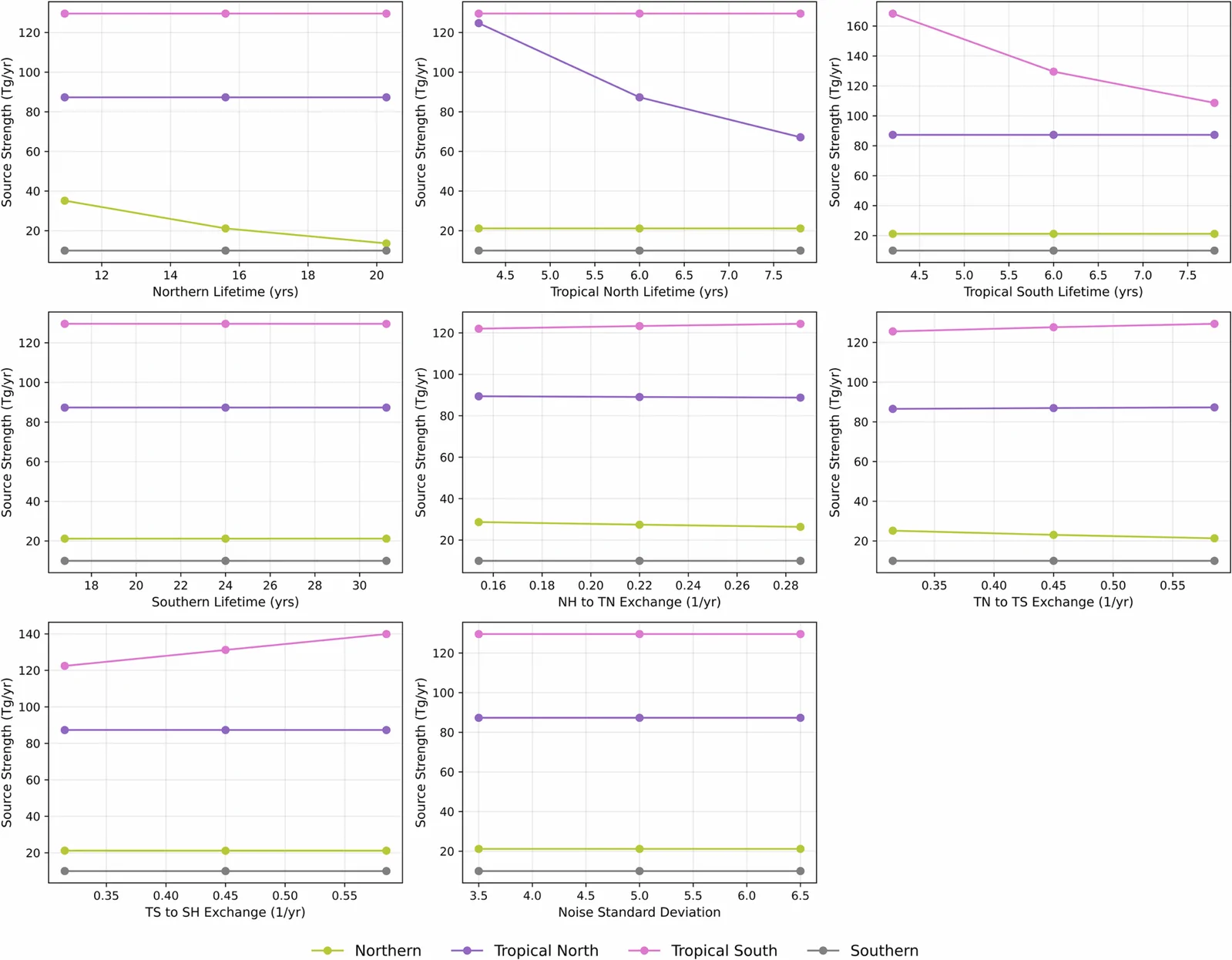
Box model sensitivity testing. Sensitivity testing outputs for the box model indicate that each latitudinal box is reasonably sensitive to the lifetime in its box. Lifetimes used in previous studies were chosen for consistency^5,51,52^. Minor sensitivity is noted in the exchange rates. Source data

Uncertainties in observations and parameters lead to uncertainties in source strength estimates from observational noise in the ice core CH_4_ concentrations, assumed atmospheric lifetimes and latitudinal exchange rates, and general structural limitations owing to the reduced complexity of the box model. We have quantified these uncertainties using a Monte Carlo approach that propagates observational (CH_4_ concentration) uncertainty, with a series of sensitivity tests that assess the influence of each lifetime and transport exchange rate (Extended Data Fig. 6; detailed in Methods). Source strength estimates are slightly sensitive to the assumed oxidation and transport parameters but even a 30% perturbation in CH_4_ lifetime and exchange rates drawn from previous literature^43,44^ produces smaller changes than in the relative latitude partitioning of source strengths from the inclusion of the SCA data. To investigate the sensitivity of the tropical north box interpolation, two more model runs were conducted using the limited data from the East Rongbuk Glacier and the tropical north data generated from the polar-only run as tropical north box inputs. These extra runs generated very slight changes (maximum 3 Tg year^−1^) in all boxes, indicating that the model is not overly sensitive to the initial interpolated tropical north box (Extended Data Table 1). We note that this model is a simplified framework of atmospheric transport and chemistry and, as such, these derived source strengths will be subject to some degree of structural uncertainty.

Previous studies have only estimated historical tropical contributions from polar records^5,7^. The addition of the SCA data confirms that fluctuations in the global CH_4_ record reflect variations in tropical sources^15^, probably wetlands. These tropical CH_4_ emissions are often considered the drivers of long-term atmospheric CH_4_ variability, with notable large-scale fluctuations during the transitions into and out of glacial and interglacial stages^5,43^. Although the surface area of low-latitude CH_4_-producing wetlands has not substantially changed in the past 300 years (ref. ^45^), climatic parameters (temperature, precipitation) have driven CH_4_ fluctuations^46^. Considering present CH_4_ production drivers^1,2^, past wetland reconstructions^42^ and previous estimates^5^, the box model results indicate a greater contribution from equatorial CH_4_ sources, highlighting low-latitude data as an important constraint for improving previous estimations^42,47,48^. The 2,000-year Huascarán SCA record fills a critical gap in historical CH_4_ records and improves our understanding of the importance of tropical sources to the global CH_4_ distribution.

## Methods

### Ice core measurements

Fifty-one discrete CH_4_ samples between 40–60 g were cut below the firn to ice transition (about 41 m) at depths ranging from 44.01 to 67.40 m (out of the total 69.33 m) in Huascarán’s SCA. The samples were shipped frozen to Oregon State University, where they were analysed for CH_4_ concentration using the standard and well-documented wet extraction procedure^5,49^. The samples were evacuated for 90 min in a vacuum flask at −60 °C in a refrigerated bath. Once evacuated, the samples were submerged in a warm water bath (50 °C) to melt the ice and release the gas into the headspace above the water. After the samples were completely melted, they were refrozen in the refrigerated bath (−60 °C). The CH_4_ concentration in the headspace was measured with a gas chromatograph^7,50^. Calculations were performed using methane peak area and headspace pressure, compared with daily measurements of calibrated air standards. The air from each sample was measured four times and the mean standard deviation of these measurements across all samples was 3.7 ppb. All of the CH_4_ concentration measurements were conducted in the same laboratory at Oregon State University, removing any potential issues for scale offsets. Not enough ice was available for replicate measurements.

Five more samples (from 47.24, 57.53, 60.76, 63.22 and 64.11 m depth) were analysed for their stable carbon isotopic composition of methane (*δ*^13^C-CH_4_) at Oregon State University following the procedure detailed in previous studies^50,51^. Ice samples for *δ*^13^C-CH_4_ analysis were sealed in a glass extraction chamber held at −60 °C while the residual air was evacuated. The sample was melted in an ultrapure helium atmosphere and the air released was transferred to an extraction line using a helium carrier^50^. A series of gas traps separated methane and other hydrocarbons from bulk air before a gas chromatographic column isolated CH_4_ from any residual gases (for example, CO and CO_2_). The CH_4_ was then oxidized to CO_2_ using a Thermo GC IsoLink interface and analysed for *δ*^13^C-CO_2_ using a Thermo Delta V isotope ratio mass spectrometer in continuous-flow mode. *δ*^13^C-CO_2_ was translated to *δ*^13^C-CH_4_ by bracketing each sample with reference air standards of known isotopic composition and presented relative to the Vienna Pee Dee Belemnite scale. Besides the ice-melting steps, air standard measurements were treated identically to those of ice samples. Data were not corrected for gravitational settling^52^ or diffusive isotopic fractionation^34^ during gas transport in the firn column, as the required data to do so did not yet exist. However, we expect these corrections to be small relative to the measured difference in *δ*^13^C-CH_4_ between SCA and other polar records. We note an expected diffusive fractionation in which CH_4_ growth rates are highest (for example, the oldest and youngest samples), data may be depleted (between 0.29 and 0.43‰ from diffusive fractionation)^34^. Sample depths were selected to correspond to PI and post-industrial revolution dates (that is, before and after 1850 CE, respectively) and sample masses used (between 130 to 300 g) were chosen on the basis of the total air content and CH_4_ concentration of the two closest samples measured during the initial CH_4_ concentration analysis. A careful determination of the sample size was important to limit linearity effects that are caused by differences in the amount of CH_4_ within each sample relative to that in each standard air measurement (7 cc of 1,900 ppb CH_4_ in air). Linearity corrections were generally lower than 0.02% and much smaller than the longer-term reproducibility of the system (±0.14‰).

**Extended Data Fig. 7:**
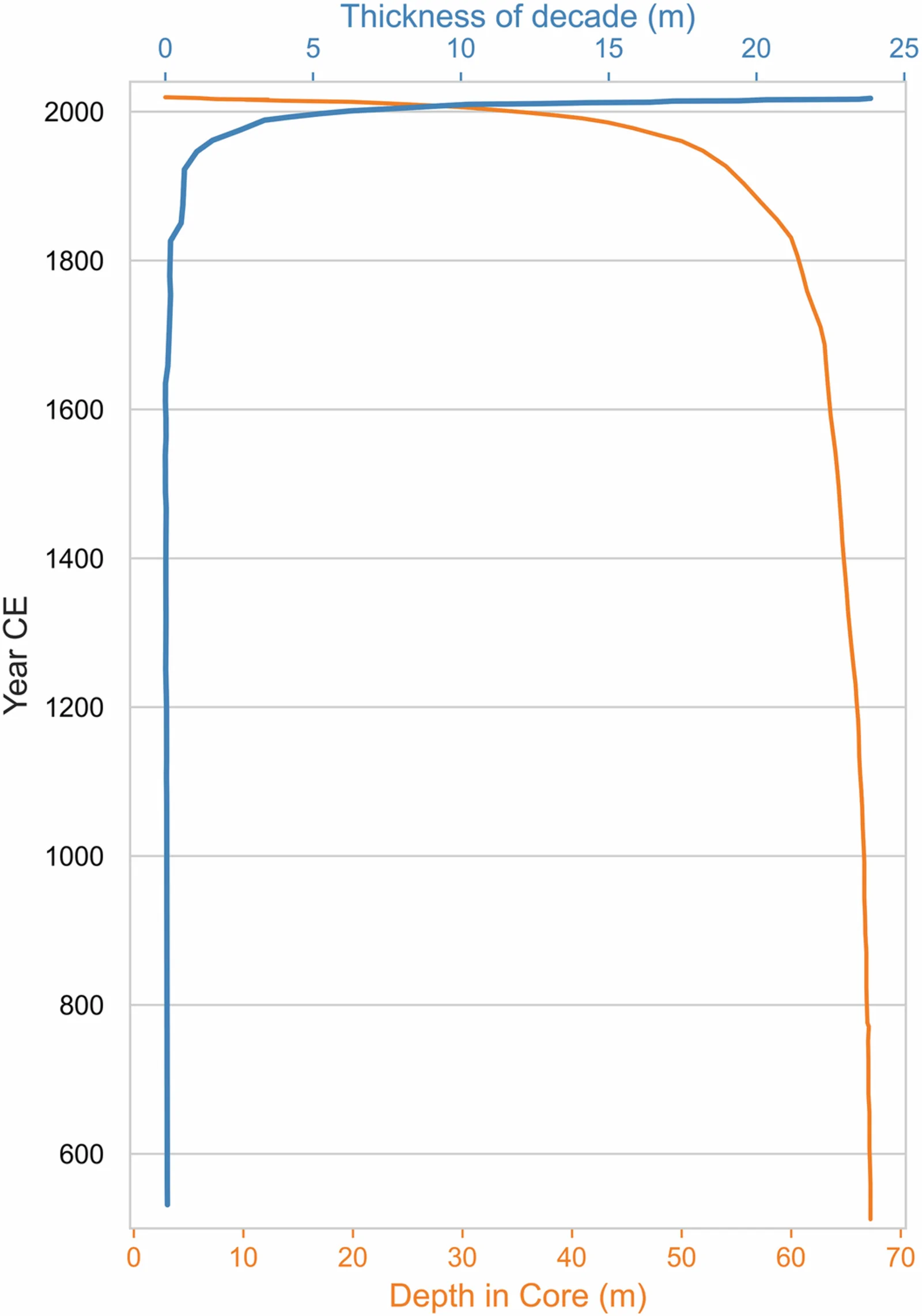
SCA layer thickness. SCA time versus depth in core (red curve) and versus the thickness of ice from each decade (blue curve), demonstrating the very rapid layer thinning before 1900 CE. Source data

All of the sample depths noted above were analysed to catalogue the past 2,000 years of CH_4_ concentration and more recent *δ*^13^C-CH_4_ fluctuations. Sampling was limited to the past 2,000 years owing to: (1) the high rate of thinning (Extended Data Fig. 7) below 55 m, which particularly limited sampling below 67.40 m, and (2) limited availability of sufficient ice below 67.40 m owing to ice required for other analyses. Ice availability was further limited for the *δ*^13^C-CH_4_ samples as a result of the low air content in SCA owing to high elevation. The sample size below 64.11 m required for the isotope analysis was approximately 380 g and the ice that remained after other analyses was not sufficient to collect a reliable sample.

### Ice core timescale and ice conditions

**Extended Data Fig. 8:**
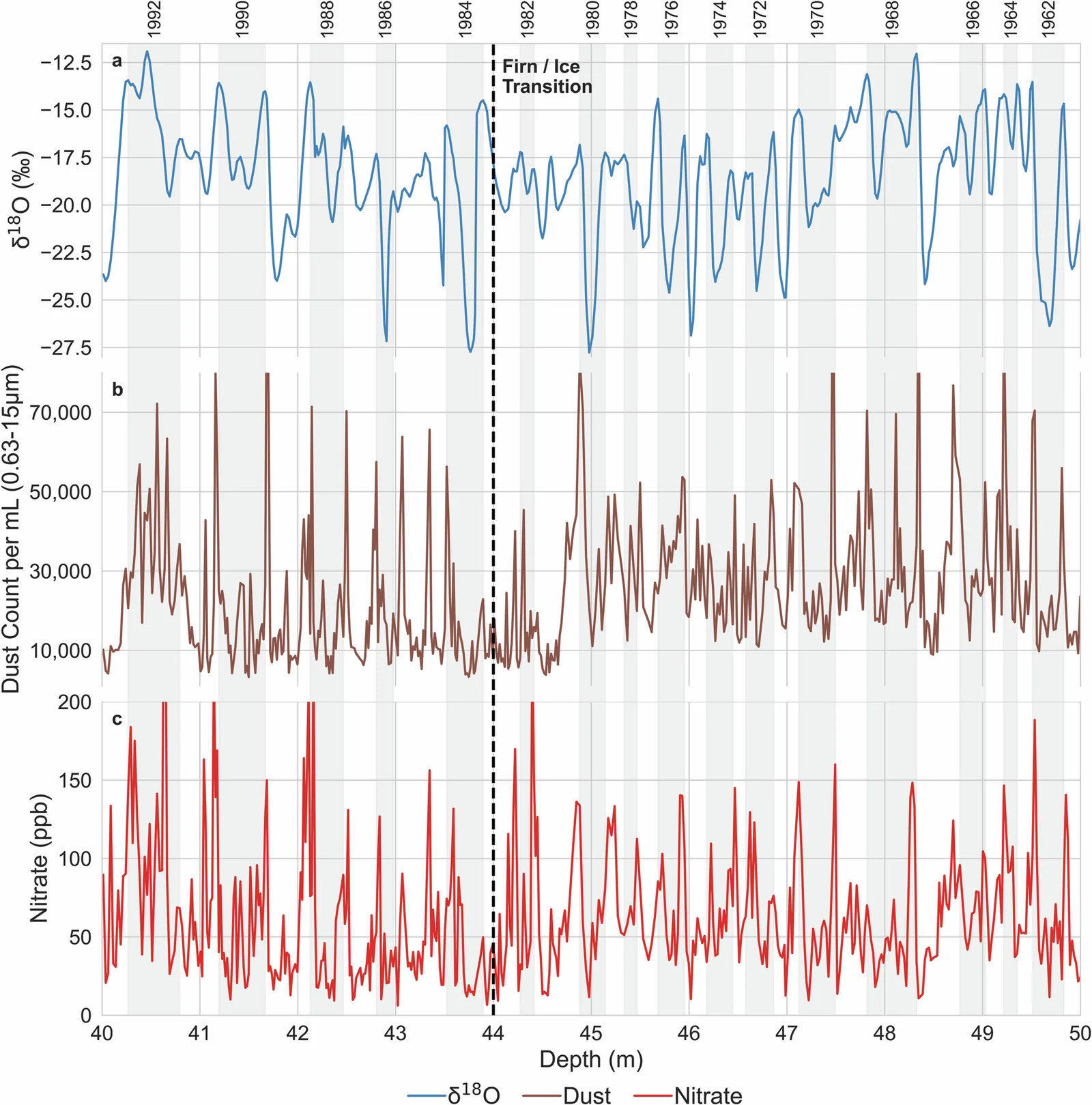
Annual layering of SCA. Annual layer counting from 40 to 50 m using SCA geochemical data showing cycles of nitrate, dust and *δ*^18^O (n = 463). Years are noted along the top *x*-axis and in alternating white and grey vertical bars and the firn to ice transition at approximately 44 m is denoted with a dashed vertical line. Source data

**Extended Data Fig. 9:**
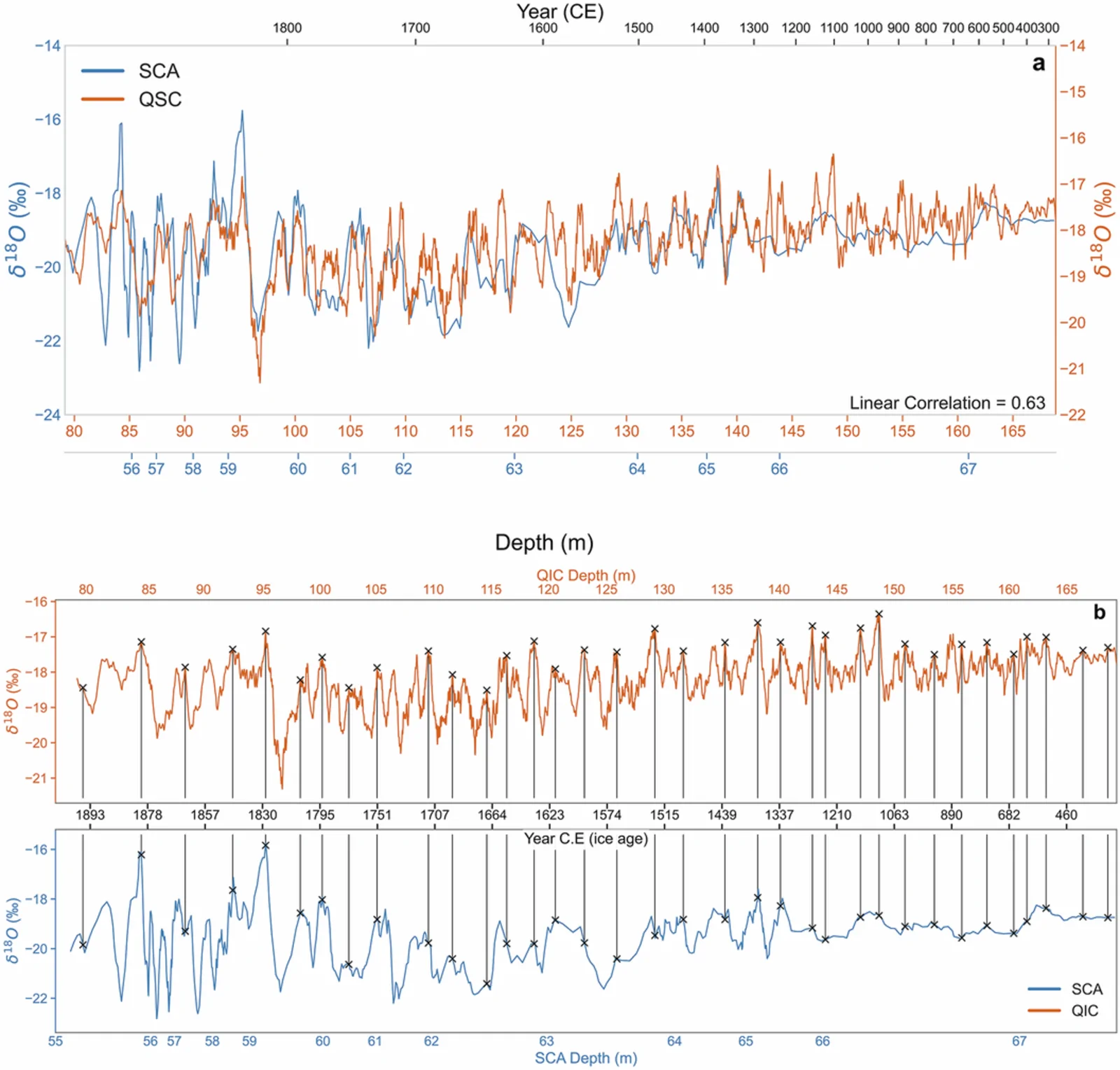
SCA timescale development. **a**,**b**, SCA timescale development before 1910 CE. AnalySeries^52^ match by depth was performed between the *δ*^18^O record from the annually dated QIC (ref. ^53^) and Huascarán SCA from 1910 CE to 300 CE. The correlation coefficient between the match is +0.63 (*P* < 0.001). The QIC and adjusted SCA records are shown overlain in panel **a** and side by side with selected tie points in panel **b**. After the depth match between the two cores was accomplished, the timescale from QIC was transferred to SCA, as shown in panel **b**. Source data

The timescale for the top 55 m of SCA (2019 to 1910 CE) was established by annual layer counting using seasonal variations in *δ*^18^O and concentrations of dust and nitrate (NO_3_^−^) to identify the annual cycles (Extended Data Fig. 8). The requirement for a year to be assigned is the alignment of at least two of the three parameters. The uncertainty in the assigned years at 55 m is ±2 years. Owing to compression, stratigraphy below 55 m thins rapidly (Extended Data Fig. 7), such that annual layer counting becomes difficult. To construct a timescale for this lower portion of SCA, the *δ*^18^O record was matched to the *δ*^18^O record from the Quelccaya summit core (Quelccaya Ice Cap, QIC) from southern Peru^53^. The QIC is located 910 km southeast of Huascarán in the Cordillera Vilcanota just above the Amazon basin. Both Quelccaya and Huascarán receive most of their precipitation from the tropical Atlantic, carried by easterlies over the Amazon, and it is reasonable to conclude that they have broadly similar *δ*^18^O profiles. Using the AnalySeries software^54^, *δ*^18^O records below the 1910 CE horizon (55.5 m in SCA, 75 m in QIC) in the two cores were matched by depth, allowing the QIC timescale to be transferred to SCA (Extended Data Fig. 9).

The linear correlation coefficient of the *δ*^18^O match between the two cores is +0.63 (*P* < 0.001). The difficulty of the matching increases with depth, increasing the potential for increasing timescale uncertainties with depth. The much higher stratigraphic thinning rate in SCA compared with QIC means that there is increasingly more time encompassed in each *δ*^18^O sample with depth relative to QIC. Also, the QIC *δ*^18^O data were smoothed with a 41-sample running mean, whereas the shorter SCA record was smoothed using a 9-sample running mean. The resulting timescale has the expected CH_4_ rise in the early 1500s, occurring between 63.9 and 64.3 m in SCA, which corresponds to approximately 1480 to 1520 CE ice age (Δage: approximately 19 years; approximately 1500 to 1540 CE gas age), which lies within the time span of the same fluctuation in polar records^7^.

The ice samples were assessed for any potential alteration that may have occurred before or after coring that could have affected the CH_4_ concentrations. After inspection, it was concluded that the SCA contains no indication of visible melt layers and all borehole temperatures recorded during drilling were between −9 and −15 °C between 0 and 10 m and around −9 °C from 10 m and below (Extended Data Fig. 4a). Following drilling and during transport, continuous temperature measurements were recorded with thermistors placed inside two randomly selected core section containment tubes. The thermistors indicate adequately low temperatures (consistent and below freezing) after drilling and throughout transport from the field to the freezer at BPCRC (Extended Data Fig. 4b,c). Furthermore, air content of SCA is consistent with what is expected on the basis of the site elevation and air content collected from other cores in both mid-latitude and polar regions (Extended Data Fig. 5). These assessments indicate that modern atmosphere has not influenced the sample concentrations^38–42^.

### Box model

Our model is based on a previously constructed four-box model used to constrain CH_4_ source distribution^5^ but adapted to use the SCA CH_4_ record as an extra constraint on the signal for the ‘tropical south’ box (0–30° S) instead of relying only on inputs for the northern and southern boxes from polar ice cores. The model consists of four boxes: northern (NH), 30–90° N; tropical north (TN), 0–30° N; tropical south (TS), 0–30° S; and southern (SH), 30–90° S. A Monte Carlo simulation (1,000 iterations) is used to vary CH_4_ inputs. True tropical boundaries are at 23.5° N and 23.5° S but 30° N to 30° S are used so that each latitudinal box has an equal air mass.

Initially, the model was run with two inputs, only polar CH_4_ data for the NH and SH boxes while solving for the TN and TS boxes using assumed constant transport fluxes. We defined a fixed lifetime for inter-hemispheric exchange and CH_4_ lifetime based on previous literature with lifetimes of 15.6 years (NH), 6 years (TN and TS) and 24 years (SH) and transport exchange rates of 0.22 years (NH–TN), 0.45 years (TN–TS) and 0.45 years (TS–SH)^43,44^. CH_4_ observations were compiled from previously published ice core records GISP2^15^ and NEEM^20^ (NH), WAIS^15^ (SH) and SCA (TS). A second model run with three inputs used the SCA record to prescribe CH_4_ in the TS box while still using the polar data for NH and SH and solving for TN. Two runs with TN inputs were conducted using the limited data from the East Rongbuk Glacier^19^ (800–1800 CE) and the synthetically generated TN data from the polar-only run for the TN box. For all model versions, we assume a total tropospheric mass of 1.78 × 10^20^ moles of dry air and use a CH_4_ molar mass of 16 g mol^−1^. To facilitate comparison, each record was interpolated onto a common 5-year age spacing based on the WAIS core using linear interpolation. Gaussian noise with a standard deviation of 5 ppb was added independently to each CH_4_ value at every time step within each iteration, representing the measurement uncertainty of the system. This perturbation was included to represent the combined analytical and sampling uncertainties in ice core CH_4_ measurements. Within the Monte Carlo framework, the observational inputs (CH_4_ concentrations) are varied across iterations, the atmospheric lifetimes and transport exchange rates are held, with their influence assessed through the sensitivity analysis described below.

The resulting source strengths (Tg CH_4_ year^−1^) in this model were calculated using the methane concentration inputs, transfer fluxes between each box and loss rate within each box. Each box is connected to adjacent ones by transport fluxes represented as first-order exchange between neighbouring boxes and all boxes are subject to loss (representing all CH_4_ sinks), with specific atmospheric lifetimes for each box. The following equation was then used to calculate output source strengths:

$${E}_{i}={\lambda }_{i}{C}_{i}+\sum _{j}\frac{1}{{T}_{{ij}}}({C}_{i}-{C}_{j})$$

to solve for *E*_*i*_ as the calculated source strength (Tg year^−1^), in which *λ*_*i*_ is the loss rate from oxidation (year^−1^), *C*_*i*_ and *C*_*j*_ are the burdens in the box and neighbouring box, respectively (Tg), *T*_*ij*_ is the transport time between these boxes (years) and Σ_*j*_ is the net transport exchange across all of the boxes (Tg year^−1^). The annual source strength is determined at each time step based on the above equation. SH source strength was fixed at 10 Tg CH_4_ year^−1^, as most studies suggest that the SH box source strengths are probably very low, have not varied and the SH box only receives transport exchange from higher latitudes^5^. All modelled results were smoothed to a 20-year average to eliminate short-term variability from any observational noise. The TN and TS outputs are summed for a ‘combined tropics’ category for discussion and visual analysis for low-latitude CH_4_ source strength. Temporal variability was calculated as the standard deviation across Monte Carlo iterations for each box at each five-year time step. Within the Monte Carlo framework, observational noise is independently resampled for each CH_4_ value for every iteration and time step, for which atmospheric lifetimes and transport exchanges are held at their set values.

Uncertainty of each source strength was quantified using the resulting ensemble spread from the Monte Carlo iterations. For each latitudinal box, the uncertainty was calculated as the standard deviation of the time-average source strength across all 1,000 simulations and is expressed as a percentage relative to the mean source strengths for that box. The average uncertainties for the three-input run are as follows: the NH box ±3.18%, the TN box ±0.50%, the TS box ±2.59% and the TS box 0%, as the source strength was hard-capped at 10 Tg year^−1^. The uncertainty is reflected in the inferred source strength magnitude from both parameter assumption and observational noise. All four run uncertainties are available in Extended Data Table 1.

Sensitivity to the lifetime and exchange rates used were conducted by varying each parameter by ±30%. Although the resulting methane source strength is notably most sensitive to lifetime uncertainty, the lifetimes used are consistent with those used in previous studies^5,43,44^. Past atmospheric chemistry model intercomparisons estimate that the CH_4_ lifetime differs by approximately 15–34% between PI and present-day conditions, as well as by a similar magnitude across models within a fixed time span^55,56^. This places our ±30% range within the uncertainty found in full chemical transport models. Transport rates between boxes are grounded in previous literature^43,44^. Model source strengths record modest sensitivity to these terms, with a maximum ±1.6 Tg year^−1^ between the NH to TN box, ±5.4 Tg year^−1^ between the TN to TS box and ±17.5 Tg year^−1^ between the TS to SH box (Extended Data Fig. 6). It should be noted that these source strengths are subject to some degree of structural uncertainty that will not be captured by the Monte Carlo approach, as this four-box model is a framework that represents a simplified explanation of atmospheric transport and the factors that affect methane lifetime. The extra runs with TN inputs derived from the East Rongbuk data from 800 to 1800 CE and the synthetically generated TN box from the polar-only run in the TN box from 0 to 1800 CE served to demonstrate that the initial interpolation of TN (the model run without TN input data) was not overly sensitive in the run with the SCA data. These four input runs resulted in minimal changes to the TN box source strength, with a maximum of 3 Tg year^−1^. The resulting source strengths are detailed in Extended Data Table 1. Overall, the changes between the polar-only model configuration and the inclusion of the SCA data are more robust in their constraints of CH_4_ distribution than they are sensitive to the interpolation of the TN data.

## Online content

Any methods, additional references, Nature Portfolio reporting summaries, source data, extended data, supplementary information, acknowledgements, peer review information; details of author contributions and competing interests; and statements of data and code availability are available at https://doi.org/10.1038/s41586-026-10938-1.

## Supplementary information

Peer Review File

## Source data

Source Data Fig. 1

Source Data Fig. 2

Source Data Extended Data Fig. 2

Source Data Extended Data Fig. 3

Source Data Extended Data Fig. 4

Source Data Extended Data Fig. 5

Source Data Extended Data Fig. 6

Source Data Extended Data Fig. 7

Source Data Extended Data Fig. 8

Source Data Extended Data Fig. 9

## Figures and Tables

**Extended Data Table 1:**
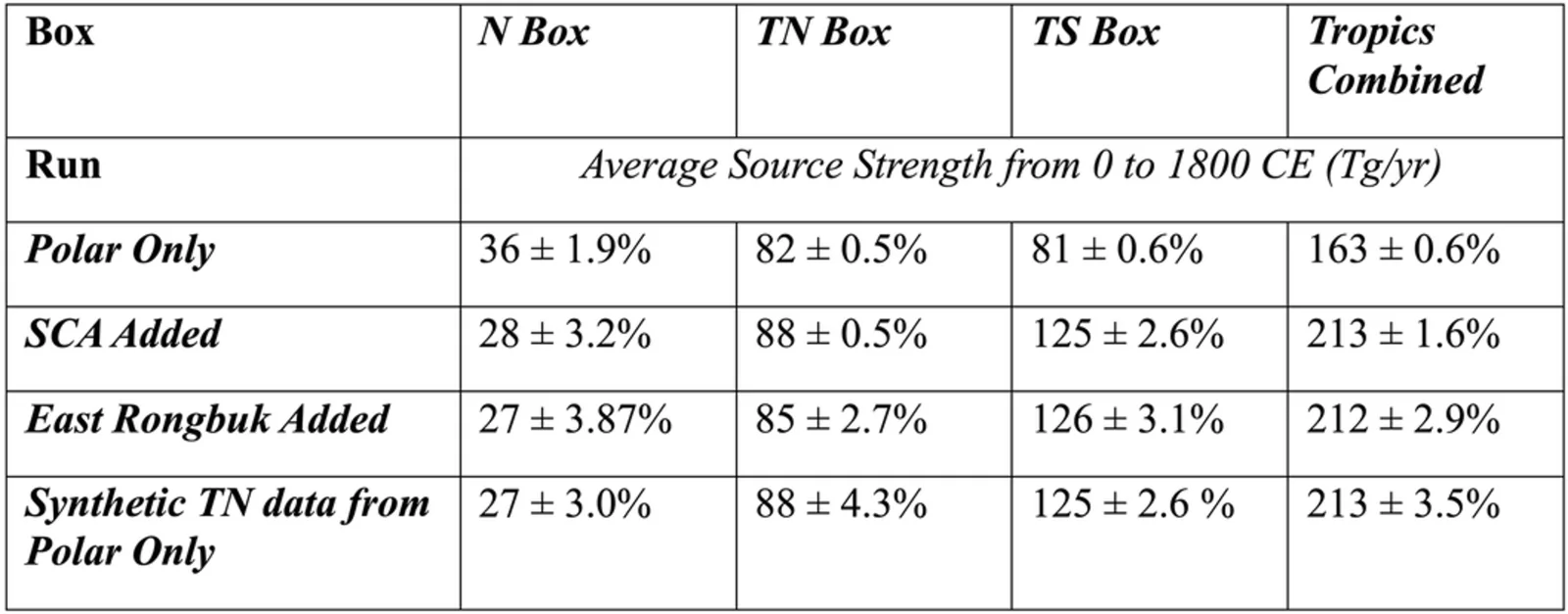
Model run box averages

Average source strength estimates for each box across each model run in Tg year^−1^ with ±standard deviations as percentages for error bounds. The combined tropics box uncertainty is reported as the average uncertainty of the two tropical boxes. The SH box was left out, as source strength there was uniformly assumed at 10 Tg year^−1^.

## Data Availability

The Huascarán SCA CH_4_ concentration and *δ*^13^C-CH_4_ isotope data are publicly available in a Zenodo repository (https://doi.org/10.5281/zenodo.18657346, ref. ^57^) as well as the NOAA National Climatic Data Center and in the NSF Public Access Repository. Source data are provided with this paper.

## Code availability

The core data used to plot Figs. 1 and 2 as well as all data used as inputs and the code used to run the four-box model are publicly available in a Zenodo repository at https://doi.org/10.5281/zenodo.18657346 (ref. ^57^).
