# Supplementary material for: A global atmospheric methane record from a tropical ice core
Source: Nature. 2026 Aug 19;657(8131):409–13. doi: 10.1038/s41586-026-10938-1 (PMC13558060; doi:10.1038/s41586-026-10938-1)
Supplement: Supplementary file 1 [file 41586_2026_10938_MOESM1_ESM.pdf]

nature portfolio

Peer Review File

A Global Atmospheric Methane Record from a Tropical Ice Core

Corresponding Author: Dr Kara Lamantia

Any redactions in this file are there to maintain patient confidentiality, the confidentiality of unpublished data, or to remove third-party material.

This file contains all reviewer reports in order by version, followed by all author rebuttals in order by version.

Attachments originally included by the reviewers as part of their assessment can be found at the end of this file.

Version 0:

Reviewer comments:

Referee #1

(Remarks to the Author)
The manuscript “A Global and Regional Atmospheric Methane Record from a Tropical Ice Core”, provides a valuable reconstruction of atmospheric methane concentrations over the past 2,000 years, based on ice cores collected at South Peak in the tropical region. This dataset helps to fill a significant gap in tropical methane measurements, providing valuable insights into regional emissions and their contribution to the global methane budget. The inclusion of δ13C-CH4 measurements further strengthens the manuscript by providing information relevant to source attribution. Overall, the study is a valuable addition to our understanding of atmospheric methane in the tropics. However, the manuscript would definitely benefit by having a clear and more explicit discussion of the four-box model’s uncertainties and limitations. Some important assumptions, such as the rates of transport between boxes and the methane oxidation, may introduce biases that are not fully captured by the Monte Carlo analysis. A more detailed discussion of the differences between uncertainty in parameters, structure and observation would help to better understand the results and show how reliable the source attribution is.

Although the Methods section describes a Monte Carlo uncertainty analysis for the four-box model and refers to a supplementary figure, the main text of the manuscript neither reports nor discusses the outcomes of this analysis. In particular, quantitative uncertainty estimates are missing from the sections presenting the model results. The authors should incorporate these results and explicitly quantify the uncertainties in their estimates.

Specific comments:

Line 37: check reference numbers, since number 5 was not cited yet.

Line 41: any reference?

Lines 73-76: What uncertainties are associated with these measurements? Looking at Figure 1, the difference between SCA and the other ice-core measurements was larger until 1700 and then became more similar, mainly to GISP2 and NEEM. Is there a specific reason for this?

Lines 120-122: Is there a specific reason for the gradual decline in the early 18th century, followed by a sharp rise from the PI to the post-1850 CE period?

Lines 132-135 and Fig.S3: Add the correlation plot between SCA CH4 and dust concentration to figure S3.

Lines 135-138: What do you mean by ‘closer’? Support your discussion by quantifying the dust and Ca^{2+} concentrations and differences.

Section: Constraining Historical CH_4 Emissions: Before discussing the results, it would be helpful for the reader to include a

brief description of the four-box model at the beginning of this section.
Lines 163-169: What uncertainties are associated with the estimated methane emissions? What are the main uncertainties associated with the four-box plot model? The authors should discuss the main uncertainties and limitations associated with their estimates.

Figure 4: shows that the addition of tropical data increased the variability of tropical south emissions, resulting mainly in a decrease from the 19th century onwards. What caused this decrease? In contrast, although the magnitude of emissions in the northern tropical region changes, the long-term trend is similar to that observed when no tropical data were used in the model. Therefore, how confident can the authors be that the increase at the end of the time series is not only due to the effect of the polar data, but is also supported by the included tropical data?

Lines 170-171: how can the authors assume that the increase is related to anthropogenic sources? Is there any supporting data that has not been mentioned? Otherwise, they should make it clear that it is a hypothesis.

Line 228-230: Although the methodology and references were previously described, it would be helpful to include a brief summary of the procedure in the Methods section.

Lines 323–330: If a Monte Carlo simulation was used to estimate the uncertainties, why were these results not used to provide a quantitative estimate of the uncertainty in the mean emissions for each region? What are the main conclusions of this analysis? For example, which parameter change had the largest effect on uncertainty in emissions?

(Remarks on code availability)

Referee #2

(Remarks to the Author)
A. Key results
The manuscript's core result is a new 2,000 year CH4 record from a high elevation tropical ice core (Huascarán SCA), which provides a rare, apparently artifact free low latitude constraint on atmospheric methane. The data show systematically higher PI CH4 relative to polar cores and, when assimilated into a four box model, lead to higher inferred tropical emissions, particularly in the southern tropics, while leaving the anthropogenic rise and high covariance with polar records intact. The confirmation of high tropical emissions is in general agreement with the present understanding of the methane budget.

B. Originality and significance
The novel contribution is the SCA dataset itself: a long, well behaved tropical ice core CH4 (and sparse 13C CH4) record that fills a geographic gap and avoids the melt/dust issues of previous tropical and subtropical cores. The four box modeling is an important use of these data but it is not fully convincing to me; the fact that constraining a third box with data improves partitioning in a four box framework is expected. This, on its own, not sufficient to justify a high impact claim. I am missing a clearer and statistically well supported articulation of the new scientific insight.

C. Data and methodology
The CH4 and 13C CH4 analytical methods are state of the art and appropriate, and the efforts to rule out in situ CH4 production or post depositional alteration (dust/Ca^{2+} correlations, melt inspection, air content–elevation comparison, borehole and transport temperatures) are thorough. In the main text, the detailed treatment of temperature and air content control (lines 142 ff, including Figures S4–S5 and the air content–elevation rationale behind Figure 3) is highly technical and could be summarized briefly in the main text, with the full description and the current Figure 3 moved to the Supplement.

D. Statistical treatment and uncertainty handling
The Monte Carlo implementation of the four box model with observational noise and reported standard deviations illustrative, but in my view the fact that only 3 boxes of the 4 box model are constrained by data is a weakness. It is know that in such evaluations models may use the unconstrained boxes in unphysical ways to build a solution. The apparent temporal behaviour of emissions in the tropical south box, including the notable decrease highlighted in the results, may partly reflect the model's structural design and the fact that two tropical boxes are only constrained by one single record with limited temporal resolution and age precision. The robustness of these temporal features should be discussed more critically. Also, I wonder whether other model structural uncertainties (e.g., lifetimes, exchange rates) are fully propagated.

E. Conclusions
As mentioned above (B and D) I am not convinced that the solution that the authors present is unique, in particular I am concerned by the fact that one box is not constrained by data. The authors do show convincingly how model results can change between having 2 and 3 boxes constrained by data. Therefore I wonder how the results would change if there were also data for the 4th box.

F. Suggested improvements
- Scientific argumentation and repetition
Streamline the narrative to avoid restating the same points (e.g., SCA uniqueness, agreement with polar records, and tropical dominance of PI emissions) across abstract, introduction, results, and conclusion; each section should add a distinct layer (motivation, methods, key results, implications) rather than recycling full arguments.

- In the “Constraining Historical CH4 Emissions” section, sharpen the main scientific message: explicitly state what is non trivial about the new constraints (e.g., the magnitude and uncertainty of PI equatorial emissions and their variability) and separate this from the more predictable improvement that comes from having an additional constrained box.

- Figures and visual presentation
I am a bit disappointed by the Figure quality. This community is know for producing high quality figures that support the narrative, but in this manuscript this is not the case. Suggestions: Merge Figure 1a and 1b into a single multi panel figure. For example, use an inset in Fig 1a to illustrated the 0–1850 CE period, but using a common, clearly labeled x axis and consistent scaling to make temporal comparisons straightforward and reduce redundancy.
Incorporate the current Figure 2 (13C CH4) as an additional panel in the same figure, aligning the timescale with the CH4 panels to visually link concentration and isotope behaviour.
- Move Figure 3 (air content vs elevation) to the Supplement and refer to it briefly in a single main text sentence as supporting evidence that air content is as expected for elevation and shows no sign of modern contamination.
- It would be nice to have a Figure with the location of the SCA site, at least the location needs to be stated precisely. I had to look it up.

Isotopic interpretation and wording
- Correct the statement around line 117: –48‰ is the approximate global atmospheric 13C CH4 value, not the weighted global emission value; the latter is more depleted because the sink fractionates strongly, so this distinction should be made explicit.
- Revise the description of the SCA 13C CH4 record (lines 120 ff): the SCA data do not clearly show a pronounced decline into the early 18th century followed by a rapid rise; rather, they are relatively constant through the PI, with the main increase occurring only after ~1850, and the text should be aligned with what is directly visible in the data.
- The reference to “Keeling plots” in the Supplement is misleading because no actual Keeling (or Miller–Tans) plots are shown; clarify what is plotted. If the goal is to infer source signatures or deviations from a background relationship, specify the underlying mathematical model (linear mixing line in concentration–isotope space, intercept definition, regression method), and state explicitly what the SCA points indicate relative to that model. Alternatively, use proper Keeling or Miller–Tans plots with regression lines and uncertainties.

Placement of technical content
- Move the very detailed technical paragraph beginning around line 142 (borehole temperatures, transport thermistors, container temperatures, air content curve details) from the main text into the Supplement and leave a concise summary in the main text indicating that temperature control and air content have been verified and support data integrity.

G. Referencing
The manuscript cites appropriate work on global CH4 budgets, previous low latitude cores, and isotopic and modeling frameworks. Some references are used in multiple contexts without clearly distinguishing when they provide data, methods, or conceptual background; clarifying these roles occasionally would improve readability. The discussion of the atmospheric versus emission weighted 13C CH4 value should also include a specific citation when the corrected interpretation is introduced.

H. Clarity of abstract, introduction, and conclusion
The abstract effectively communicates the existence and importance of the new tropical dataset. The end of the abstract "it confirms the consistent dominance of tropical emissions" is the main scientific result, which is not really unexpected.
The introduction is well motivated but somewhat repetitive regarding the problems with previous tropical cores. Condensing this discussion and more sharply highlighting the unique value of the SCA record would strengthen the argumentation. The conclusion could be tightened to focus on a small set of clearly stated, data driven advances: unique tropical record, quantitative increase in inferred PI tropical emissions, improved but still uncertain latitudinal partitioning. and to tone down any over interpretation of model dependent intra box temporal structure

(Remarks on code availability)

Referee #3

(Remarks to the Author)
This manuscript presents a 2,000-year CH_4 concentration and $\delta^{13}C$-CH_4 record from the Summit Core A (SCA) ice core drilled at Nevado Huascarán, Peru. The authors argue that this record constitutes the first reliable long-term tropical ice core CH_4 record, bridging a known spatial gap between Greenland and Antarctic records. They demonstrate the relationship between the SCA record and specific polar cores during global transitions (anthropogenic and climatic), and show agreement with the Mauna Loa instrumental record. Incorporation of the tropical record into a four-box atmospheric model yields higher inferred tropical methane emissions than estimates based solely on polar data, reinforcing the long-standing hypothesis that low latitude wetlands dominate preindustrial methane variability.

The recovery of a 2000-year tropical ice core CH_4 record is a notable achievement. Previous low-latitude records such as Dasuopu have been compromised by melt, dust-related excess methane, or large analytical uncertainty, limiting their

interpretability. In this context, the SCA record is significant; it provides a direct constraint on tropical methane variability. Where the authors contextualize this study with prior modelling and ice core studies, they may wish to more explicitly delineate how their conclusions extend beyond earlier box-model reconstructions of tropical dominance.

The analytical methods for CH_4 concentration and isotopic measurements are well established and described (I would suggest some of these detailed, standard methods go in the supplement). The ice core dating, particularly below the annual layer counted portion of the record, needs to be fleshed out. I am not convinced of the dating methods nor accuracy in the deepest part of the record, as very little is shared regarding the oldest part of the developed chronology. I would certainly like to see the glaciochemical data for an annual layer counted section of the record, as well as more information and perhaps a figure (in the supplement) regarding the deepest part of the age scale. The authors do address commonly known complications affecting non-polar ice cores (e.g., dust-driven in situ production, melt layers), and the absence of visible melt layers, low dust and Ca^{2+} concentrations, consistent air content, and agreement with modern atmospheric observations together support the integrity of the record. But again the reliance on CH_4 pattern matching in deeper sections is circular and needs to be addressed.

Uncertainties in concentration measurements are reported and propagated through the Monte Carlo simulations of the four-box model. I believe the use of ensemble simulations to account for observational noise and parameter uncertainty is appropriate. However, a clearer discussion of structural uncertainties in the box model itself, particularly the sensitivity of inferred tropical source strengths to the assumed inter-hemispheric exchange rates, the box boundaries, and prescribed OH lifetimes, is warranted.

The central conclusions (that the SCA record reflects a global atmospheric signal with a strong equatorial imprint, and that tropical emissions dominate preindustrial methane variability) are supported by the data. Some statements could be tempered to distinguish more clearly between well-supported qualitative insights and model-dependent numerical values. Overall I found notable redundancy in the text which could be tightened, with statements being repeated throughout. With a note that modelling is not my own area of expertise, it seems that model sensitivity could be clarified perhaps by a sensitivity analysis in the supplement.

The final note here is that the isotopic dataset is terribly sparse (five samples). While understandable given ice availability, the authors should explicitly discuss how this limits temporal interpretation and whether additional measurements could refine source attribution. It seems clear that this study could be greatly strengthened by more data, and efforts should be made to accommodate this. The figures could use refinement, particularly those combining multiple ice core records (e.g., clearer legends, indication of smoothing or averaging windows).

(Remarks on code availability)
I downloaded and am currently working to run the code in Python, but want to send my review without further delay. This is not my strong suit so may take me a bit to run successfully.

Version 1:

Reviewer comments:

Referee #1

(Remarks to the Author)
This study's main novelty comes from the tropical ice-core measurements, with the Huascarán SCA record filling a critical gap in historical methane records. The authors have satisfactorily addressed the major concerns raised in my original review. This is particularly evident in the clearer discussion of the uncertainties and limitations of the four-box model, as well as in the addition of sensitivity analyses that explore the effects of methane lifetime and inter-latitudinal exchange rates on the results. They have also appropriately addressed the remaining minor comments, thereby improving the clarity and presentation of the manuscript further. Overall, the revised version is more robust, and I therefore recommend publication.

(Remarks on code availability)

Referee #2

(Remarks to the Author)
The authors have improved the manuscript, especially the figure presentation is much more clear now, and results of a sensitivity study are included, thanks for that. However, the uncertainties are not sufficiently discussed in the main paper, and they may actually question the validity/significance of the inferred emission trends, see below.

A. Summary of the key results
B. Originality and significance: if not novel, please include reference
The key results are the data from the tropical ice core themselves, and the fact that, according to the argumentation of the authors, they imply larger tropical emissions than inferred without such tropical data.

C. Data & methodology: validity of approach, quality of data, quality of presentation
D. Appropriate use of statistics and treatment of uncertainties

E. Conclusions: robustness, validity, reliability
As mentioned in my first review, upwards revisions in the tropical sources are as such not a strongly surprising result, but it is great that/if novel data that may quantitatively constrain such a revision are now available. With this in mind, it is crucial to discuss uncertainties in inferred emission rates in more detail in the main manuscript. The authors have added a sensitivity study to investigate the uncertainty space (last figure in the extended data), but in the text this is not well discussed. I acknowledge that such an uncertainty investigation is difficult in the absence of many input data, but to me in the present form the discussion of uncertainties is not strong and importantly the uncertainties are not discussed well in light of the key results. One important result from Fig E9 is that the tropical emissions are quite strongly dependent on the assumed tropical lifetimes. In the main paper, the authors conclude that emissions in the tropics need to be adjusted in the tropics, but could a similar change not be achieved with changes in the tropical lifetimes? Or partly emissions and partly lifetimes? This is actually what I understand from Fig E9: If the lifetimes in the tropical boxes were higher, smaller emissions would be needed (which makes sense) and then the inferred increase in emissions that is reported in the main text would be less, potentially far less. Thus, the reported changes in the emissions are only one way to close the budget in the four box model. This questions to some degree the validity, but at least the uncertainty, of the key scientific results reported in the main text. At the least, this has to be discussed clearly, and if other scenarios can strongly alter the tropical fluxes this has to be included in the uncertainty discussion in the main text.
The quality of the presentation is improved, but for me there is still way too much repetition, in particular sentences stating that the new data present a contamination free tropical record, or that previous studies did not have tropical data. I think the paper can still be streamlined and shortened quite a lot.

F. Suggested improvements: experiments, data for possible revision
A proper discussion of uncertainties and the robustness of the main results presented is still the main weakness. I would also still suggest that the authors try to investigate in more detail the uncertianties related to having one “empty” box. One possibility would be to provide the concentrations that are returned by the model in the present configuration for the Northern Tropics, and then run the model with synthetic input values that are different from this value, to see how sensitive the model is to the absence of NT measurements. Or add the resutls from the Himalaya glacier??
It may also be useful to add a figure of panel with the average PI concentrations (and uncertainty) and emissions (and better described uncertainty estimate) in addition to the time series, in a way that illustrates the significance of the results in a more intuitive way.

G. References: appropriate credit to previous work?
Yes

H. Clarity and context: lucidity of abstract/summary, appropriateness of abstract, introduction and conclusions
Title:
Here and elsewhere in the manuscript: "Global and regional" is confusing, remove here and change to tropical elsewhere.
Abstract:
L16: what does “its extent and productivity” refer to?
L21-22: You do not really show this (Amazon basin), it is presented in the text as hypothesis. This sentence can be left out in the abstract
Main text:
L32: why not mention the present atmospheric value instead of the one from polar cores?
L33-34: reword, and note that you here also mention desctruction, which you do neglect in your analysis (main point)
L48-49: If 15 valid points are available from the Himalaya glacier, how do thye compare to your data. Could they be used to constrain your NT box?
L74: global--> tropical
L77: Why “have the potential”?
L83: Unclear sentence: What do you mean by changes?
L97-8: Can be left out, same and more information in next sentence
L104-6: Looking at Fig 1 is is evident that only the first d13C point is very much lower than from other records, the others are far less than 1per mill lower, taking averages (especially over the strong trend from other locations) is statistically not a good idea! Report the offsets of the individual points relative to the previous records?
L154-6: This is the – in my view biased – summary explanation of the sensitivity study. This part needs to be extended a lot, and the robustness of the signal must be demonstrated better.
L172: “both a global and regional”   tropical
L175-180: Half of the sentence is repeated twice….. remove second sentence
L335-6: Mention the possible effect of firn fractionation for the deepest and most depleted sample in the main text?
L408: The variables MUST be explained here!
Fig 1: I would at least note that in panel B the SCA CH4 is LOWER than at NEEM, in contrast to the PI period.

(Remarks on code availability)

Referee #3

(Remarks to the Author)
I thank the authors for their thorough and constructive response to the initial round of reviews. The revisions have improved the manuscript in several important ways, particularly in terms of clarity, figure presentation, and expanded discussion of model uncertainty and sensitivity. The dataset remains a valuable and technically impressive contribution, providing an

important tropical constraint on atmospheric CH_4 variability. Below, I focus on the extent to which the key concerns raised in my initial review have been addressed.

Chronology and age model
The authors have made a clear effort to strengthen the description of the chronology, including the addition of new supplementary figures showing glaciochemical data and a section of annual layer counts. This additional material is helpful and represents a meaningful improvement. I remain concerned about the tuning of the deepest portion of the age model; the chronology at depth still relies on alignment to known CH_4 features which is standard. However, those same features are later used to support the interpretation of a globally coherent signal, so there's still a circularity problem here. I encourage the authors to more explicitly distinguish which components of the chronology are independently constrained versus tuned, and to more clearly communicate the uncertainty associated with the oldest part of the record (new extended data fig 8 should indicate uncertainty in this portion of the chronology).

Data integrity and glaciochemical context
The authors continue to make a convincing case that the SCA record is not significantly affected by melt, dust-driven in situ CH_4 production, or other common issues in low-latitude ice cores. The addition of supporting figures and expanded methodological detail strengthens this aspect of the manuscript.

Treatment of uncertainties and model framework
This is an area where the manuscript has improved. The authors have now included a more explicit discussion of uncertainty sources (observational, parametric, and structural) in both the main text and methods, and have added a sensitivity analysis exploring the effects of transport rates and atmospheric lifetimes. The identification of OH lifetime as the dominant sensitivity is useful.
I still find that the treatment of structural uncertainty in the four-box model remains somewhat limited. While the sensitivity tests are helpful, they do not fully address concerns about model design. As raised previously (and by other reviewers), this leaves open the possibility that aspects of the inferred latitudinal partitioning may reflect model structure rather than uniquely data-driven constraints. The authors may wish to more explicitly acknowledge this limitation in the main text and to temper interpretation of model-derived temporal variability accordingly.

Isotopic constraints
The authors have clarified the interpretation of the $\delta^{13}C$-CH_4 data and corrected several issues in wording. They have also now explicitly acknowledged the limitations imposed by the small number of samples. However, the dataset remains extremely sparse, and this fundamentally limits its interpretive power. While I appreciate the logistical constraints (ice availability), caution in how strongly these data are used to support source attribution is necessary considering these study constraints.

Figures and presentation
The revisions to the figures are a clear improvement. The consolidation into multi-panel figures, inclusion of uncertainties, and relocation of highly technical material to the Supplement all enhance readability and presentation. There remain sentence fragments, comma misuse, and other minor errors throughout.

Conclusions and framing
The manuscript's central conclusions remain largely unchanged: the SCA record supports a strong tropical contribution to atmospheric CH_4 and suggests that previous polar-only approaches underestimated low-latitude emissions. The authors have made efforts to better articulate this point. However, as noted in my initial review, these findings primarily reinforce existing understanding rather than fundamentally revising it. While the new dataset is valuable and important, I encourage the authors to ensure that the strength of their claims remains well aligned with what is directly supported by the data versus what emerges from model-based interpretation.

Overall assessment
The manuscript has improved following revision and presents an important and unique dataset. Several of the concerns raised in the initial review - particularly regarding clarity, uncertainty discussion, and presentation - have been addressed. Some key issues remain only partially resolved, most notably the robustness and independence of the deepest chronology, the sample constraints, and the structural limitations of the four-box model and their implications for interpretation. These do not invalidate the study, but they do warrant continued caution in interpretation and clear acknowledgment of limitations.

(Remarks on code availability)

Version 2:

Reviewer comments:

Referee #2

(Remarks to the Author)
Thanks for considering the suggestions in preparing a new version of the manuscript. The scientific robustness of the manuscript has strongly improved, in particular the description of uncertainties and framing of the results is now adequate in my view.

I find the language and sentence constructions sometimes still hard to follow. Some sentences are incomplete, (too) long, sloppy or grammatically wrong. Nominal writing style could be exchanged to verbal style at several places to improve readability. Few examples for clarifying language are included below, but there are more language issues that should be improved. Please review carefully among the authors.

Check the use of “equatorial” versus “tropical”. For the site, it may be adequate to refer to it as equatorial, but for sources tropical may be the better term.

It is hard to find in which parameters are actually varied in the 1000 members of the Monte Carlo simulations. The main paper mentions “parameter uncertainty and concentration variability”. Line 831 mentions 5 ppb concentration uncertainty. Lines 825/26 mention life times and exchange times, are these also varied, and over which range? If not, which other parameters are varied? It would be useful to summarize this clearly at some place.

Spoecific comments (line numbers refer to “track changes” document on server)
1) Line 30-31: Thanks for including the current atmospheric values, but is this correct? Please have a look at https://gml.noaa.gov/ccgg/trends_ch4/. We are not at 1964 ppb yet as global average, and the 2024 value seems to be about 1925 ppb. From the title of the cited reference it sounds that the value derived there is for the NH (only NH stations are mentioned). I suggest using the global average value in this introduction. Also, the 800 ppb maximum cited for the past 800,000 years is not adequate as value for year 1750 when assessing the increase from 1750-present. Meinshausen et al (Table 6) state 731 ppb for year 1750. Using the more adequate numbers the increase since 1750 is > 160%.
2) Line 89: consider a different word than “reference”, maybe simply “record”. Reference has often more specific meaning in science. Also, in the description of Fig 1, I miss the most obvious feature that SCA is higher than the polar cores in the PI period. It only comes in line 98, consider moving this up?
3) Line 97: just by eye, the NEEM-SCA difference seems to be much larger than 20 ppb, at least in the latter part. Make sure you specify the period and check the value, in order not to confuse readers (it is confusing to me).
4) Line 119: please find a better construction than “global fluctuation similarity”
5) Line 259: Difficult to follow the argumentation: I assume you want to say that this demonstrates that the observed changes in concentrations must be to first order driven by source changes.
6) Line 260: Incomplete sentence. Conducted to remove? The entire sentence could be clarified
7) Line 265: …the derived source strengths are …
8) Line 268: …. contributions from polar records only.
9) Line 276:…. from equatorial CH4 sources.
10) Final sentence: This sounds to me like an opening sentence describing the potential. I suggest to modify this final sentence to: The 2,000-year Huascaran SCA record fills a critical gap in historical CH4 records and improves our understanding of the importance of tropical sources to the global CH4 distribution.
11) Line 490: Core
12) Line 511: Is the bath refrigerated? Please reword.
13) Line 514: remove “in”
14) Line 569: Why did you remove the per mill sign?
15) Line 630: Do you mean little ice age?
16) Line 654: Can you specify the “defined transport fluxes”? I suggest to move the information from Line 825/26 up to line 654.
17) Line 659: I would say the 16 g/mol for molar mass of methane is not an “assumption”
18) Line 911: Backed by
19) Line 919: in the with ??
20) Line 921: “as well as less in the other boxes” – rewrite
21) Line 922: Unclear sentence, …with polar only to the inclusion of the SCA data -- ??, also the last addition, please rewrite and read carefully!!

(Remarks on code availability)

Referee #3

(Remarks to the Author)
I thank the authors for their careful and thorough response to my second-round comments. The revisions represent a meaningful improvement to the manuscript across several dimensions. The chronology section has been substantially reworked. The decision to anchor the deep portion of the age model to the Quelccaya summit ice core rather than CH_4 features directly addresses my concern about circularity, and the additional Extended Data figure showing this alignment is a strong addition. The uncertainty associated with the deepest portion of the record is now more clearly communicated. The expanded uncertainty treatment is appreciated. The authors have appropriately rewritten the uncertainty discussion in both the main text and methods, now explicitly identifying observational, parametric, and structural sources of uncertainty. The addition of the two supplementary model runs effectively demonstrates that the model is not overtly sensitive to the interpolated tropical north box, addressing a concern raised across multiple rounds of review. The Monte Carlo propagation approach and the demonstration that even a 30% perturbation to lifetimes and transport rates produces changes smaller than the effect of adding the SCA record together provide a reasonable case for the robustness of the main findings. I note that structural uncertainty in the four-box model remains an inherent limitation of this study, and some aspects of the inferred latitudinal partitioning may still reflect model design. However, the authors have now acknowledged this explicitly and have

appropriately tempered their conclusions. The revised closing sentence accurately reflects what the data directly support. The isotopic data remain sparse, and the authors have acknowledged this limitation along with the potential influence of firn fractionation. The interpretation I would say is now suitably cautious. The figures, presentation, and editorial formatting all meet the required standards. I am satisfied that the authors have addressed my concerns to the extent feasible given the available data, and I recommend acceptance for publication. Alison Criscitiello

(Remarks on code availability)

Open Access This Peer Review File is licensed under a Creative Commons Attribution 4.0 International License, which permits use, sharing, adaptation, distribution and reproduction in any medium or format, as long as you give appropriate credit to the original author(s) and the source, provide a link to the Creative Commons license, and indicate if changes were made.
In cases where reviewers are anonymous, credit should be given to 'Anonymous Referee' and the source.
The images or other third party material in this Peer Review File are included in the article's Creative Commons license, unless indicated otherwise in a credit line to the material. If material is not included in the article's Creative Commons license and your intended use is not permitted by statutory regulation or exceeds the permitted use, you will need to obtain permission directly from the copyright holder.
To view a copy of this license, visit https://creativecommons.org/licenses/by/4.0/

We would like to thank all the reviewers for their comments on our manuscript. All our responses are recorded in blue text. While a few comments were similar from each individual reviewer, we have written our responses in all locations of the comment to ensure everything was fully addressed. Line references are for the document with all changes accepted/ tracked changes turned off.

Referee #1 (Remarks to the Author):

The manuscript "A Global and Regional Atmospheric Methane Record from a Tropical Ice Core", provides a valuable reconstruction of atmospheric methane concentrations over the past 2,000 years, based on ice cores collected at South Peak in the tropical region. This dataset helps to fill a significant gap in tropical methane measurements, providing valuable insights into regional emissions and their contribution to the global methane budget. The inclusion of δ13C-CH4 measurements further strengthens the manuscript by providing information relevant to source attribution. Overall, the study is a valuable addition to our understanding of atmospheric methane in the tropics. However, the manuscript would definitely benefit by having a clear and more explicit discussion of the four-box model's uncertainties and limitations. Some important assumptions, such as the rates of transport between boxes and the methane oxidation, may introduce biases that are not fully captured by the Monte Carlo analysis. A more detailed discussion of the differences between uncertainty in parameters, structure and observation would help to better understand the results and show how reliable the source attribution is.

Although the Methods section describes a Monte Carlo uncertainty analysis for the four-box model and refers to a supplementary figure, the main text of the manuscript neither reports nor discusses the outcomes of this analysis. In particular, quantitative uncertainty estimates are missing from the sections presenting the model results. The authors should incorporate these results and explicitly quantify the uncertainties in their estimates.

Specific comments:

Line 37: check reference numbers, since number 5 was not cited yet.

Line 18: References added to the summary include reference 5

Line 41: any reference?
Line 17-18: References added and this sentence was moved and rephrased into the summary instead of in the main text

Lines 73-76: What uncertainties are associated with these measurements? Looking at Figure 1, the difference between SCA and the other ice-core measurements was larger until 1700 and then became more similar, mainly to GISP2 and NEEM. Is there a specific reason for this?
Line 65-67: We have added specification that the difference after the Pre-industrial period changes, as the difference between polar cores that extend far enough past the pre-industrial (NEEM and Law Dome) experience a larger ppb difference.
Line 70-72: There are two SCA measurements that dip slightly lower (in range of the Greenland core) around 1700 or so, but we did not want to make any large claims about two data points. The margin of

error for all of these measurements could result is a slight disparity of about 7 ppb as well. Post PI the cores do push slightly more together as the human induced increase takes over and overwhelms all the signals.

Figure 1: We have added the uncertainties to the figure 1 caption (Line 290-298).
Other adjustments have been made to Figure 1, including the following: Figures 1 and 2 have been merged as suggested into Figure 1. We have re-organized Figure 1 and 2 into a 3 panel figure with a shared x axis and differing y axes to display the changes in the record, with an initial focus on the pre-industrial period, and then a more zoomed out view of the concentrations with the anthropogenic spike highlighted by the core data and the Mauna Loa record. The d13C data (previously Figure 2) is plotted below the concentration data on the same axis as Figure 1c.

Lines 120-122: Is there a specific reason for the gradual decline in the early 18th century, followed by a sharp rise from the PI to the post-1850 CE period?
Line 102-103: Yes, the anthropogenic influence on the d13C signature moves it to less negative values. We have specified this in the text that the changing signal is likely from fossil fuel production as follows: "The overall trend in the SCA $\delta^{13}C$-CH_4 record mirrors that of existing polar records, which have been characterized by depletion into the early 1800s followed by a sharp enrichment post 1850 CE (a likely shift to a more fossil fuel based signature[7]) and therefore support that SCA is capturing a global methane signal."

Lines 132-135 and Fig.S3: Add the correlation plot between SCA CH4 and dust concentration to figure S3.
We have added correlation plots with regression lines between SCA CH4 and dust and SCA CH4 and calcium into the supplement figure (now Extended Data Figure 3).

Lines 135-138: What do you mean by 'closer'? Support your discussion by quantifying the dust and Ca^{2+} concentrations and differences.
Line 121-122: After looking at the appropriate references and databases, they do not explicitly list the dust levels but only note that they are low and not an issue to the methane record. We have reworded this sentence to align with what is published in the references as follows: "The dust and Ca^{2+} concentrations are also orders of magnitude lower than in other cores in which dust is likely responsible for excess CH_4 production (e.g., Sajama in Bolivia[16] and the NGRIP Greenland core[17]) and likely align better with Antarctic cores in which excess CH_4 from dust is noted as not being an issue[28,37]."

Section: Constraining Historical CH_4 Emissions: Before discussing the results, it would be helpful for the reader to include a brief description of the four-box model at the beginning of this section.
Line 129-135: We have added a description of the model as requested to the beginning of this sentence. The text reads as follows: "To better constrain PI CH_4 latitudinal emission distribution, we applied a four-box atmospheric model with the intention of partitioning sources across each of the four latitude bands. In previous implementations, only polar ice cores have been available and tropical emissions remained implicitly inferred through hemispheric transport assumptions rather than having a direct observational constraint. These polar-only studies highlight the historical dominance of natural emissions in the tropical latitudes[2,5,7], with one such study specifically using polar ice core data and a global box model to estimate tropical emissions[5]. *"*

Lines 163-169: What uncertainties are associated with the estimated methane emissions? What are the

main uncertainties associated with the four-box plot model? The authors should discuss the main uncertainties and limitations associated with their estimates.
Line 149-156: Uncertainty is discussed here in the main text as follows: "Uncertainties arise in the source strength estimates from observational noise in the ice-core CH_4 concentrations, assumed atmospheric lifetimes, latitudinal box exchange rates, and general structural limitations from the box model's reduced complexity in comparison to the global atmosphere. We have quantified these uncertainties using a Monte Carlo approach that propagates parameter uncertainty and concentration variability as well as sensitivity testing (detailed in methods). While source strength estimates are somewhat sensitive to the assumed oxidation and transport parameters, the enhancement of the tropical southern source strength following the addition of the SCA record is robust to variability from these inputs."

More detail is below regarding specifics in the methods section.

Line 426-450: Uncertainty is now discussed in detail in this section in the methods. We specify the Monte Carlo method here, report each box's average uncertainty in a ± % value and discuss general variability in each box's signal that results from the Monte Carlo method. We have also run sensitivity tests and report the necessary results in here that indicate while the OH loss rate is naturally the largest sensitivity; we use reported values from other similar studies to stay consistent as our goal was to show that the addition of a low latitude core record is important to underestimating historical methane fluctuations. We make the case for the reliability of our results but also note that this box model is simplified relative to the global atmosphere and thus it is expected some limitations will occur.

Figure 4: shows that the addition of tropical data increased the variability of tropical south emissions, resulting mainly in a decrease from the 19th century onwards. What caused this decrease? In contrast, although the magnitude of emissions in the northern tropical region changes, the long-term trend is similar to that observed when no tropical data were used in the model. Therefore, how confident can the authors be that the increase at the end of the time series is not only due to the effect of the polar data, but is also supported by the included tropical data?
Overall, the model is a simplified version of the global atmosphere and as odd changes start to occur around the industrial revolution, we believe that a more complex model would be necessary to better constrain all the latitudinal bands more appropriately. We have chosen to cut Figure 2 (previously Figure 4) and the model off at 1800 CE. The main purpose of this model was to identify that previous historical estimates were undercutting the tropical contribution because we did not have a good low latitude record and everything was being interpreted from polar cores. The addition of the SCA record shows that the Tropical south box emissions are necessary for better interpretation. This manuscript is largely concerned with pre-industrial time periods due to the nature of the record and past ice core records for the purposes of better understanding historical fluctuations and so by cutting the model at 1800, that will remain the focus.

We note this on Line 144-146 to account for the above and below comment: "While the model is halted at 1800 CE due to uncertainties related to rapidly changing concentrations, we hypothesize anthropogenic production of CH_4 would create increases in the tropical north and northern boxes."

Lines 170-171: how can the authors assume that the increase is related to anthropogenic sources? Is there any supporting data that has not been mentioned? Otherwise, they should make it clear that it is a hypothesis.
As this is also related to the above comment, this section has been changed to what is reported above on Line 144-146 discussing potential outcomes past 1800 CE. As we are mostly focused on historical and

pre-industrial changes we chose not to model past 1800 in order to involve a lot of complications that would likely arise with the addition of anthropogenic emission dynamics and that is largely out of scope for this manuscript.

Line 228-230: Although the methodology and references were previously described, it would be helpful to include a brief summary of the procedure in the Methods section.
This comment was about mentions of the d13C data, we have reorganized the ice core measurement section to discuss the sampling methodology with the appropriately ice selection process. All method details for d13C are in Lines 320-344.
Lines 323–330: If a Monte Carlo simulation was used to estimate the uncertainties, why were these results not used to provide a quantitative estimate of the uncertainty in the mean emissions for each region? What are the main conclusions of this analysis? For example, which parameter change had the largest effect on uncertainty in emissions?
Uncertainty is now discussed in detail in this section in the methods and we have included a sensitivity analysis as well. From Line 426-450 we specify the Monte Carlo method here, report each box's average uncertainty in a ± % value and discuss general variability in each box's signal that results from the Monte Carlo method. We have also run sensitivity tests and report the necessary results in here that indicate while the OH loss rate is naturally the largest sensitivity; we use reported values from other similar studies to stay consistent as our goal was to show that the addition of a low latitude core record is important to underestimating historical methane fluctuations. We make the case for the reliability of our results but also note that this box model is simplified relative to the global atmosphere and thus it is expected some limitations will occur.

Referee #2 (Remarks to the Author):

A. Key results
The manuscript's core result is a new 2,000 year CH4 record from a high elevation tropical ice core (Huascarán SCA), which provides a rare, apparently artifact free low latitude constraint on atmospheric methane. The data show systematically higher PI CH4 relative to polar cores and, when assimilated into a four box model, lead to higher inferred tropical emissions, particularly in the southern tropics, while leaving the anthropogenic rise and high covariance with polar records intact. The confirmation of high tropical emissions is in general agreement with the present understanding of the methane budget.

B. Originality and significance
The novel contribution is the SCA dataset itself: a long, well behaved tropical ice core CH4 (and sparse 13C CH4) record that fills a geographic gap and avoids the melt/dust issues of previous tropical and subtropical cores. The four box modeling is an important use of these data but it is not fully convincing to me; the fact that constraining a third box with data improves partitioning in a four box framework is expected. This, on its own, not sufficient to justify a high impact claim. I am missing a clearer and statistically well supported articulation of the new scientific insight.
We have addressed these notes below in the reviewer's suggested improvement section

C. Data and methodology
The CH4 and 13C CH4 analytical methods are state of the art and appropriate, and the efforts to rule out in situ CH4 production or post depositional alteration (dust/Ca^{2+} correlations, melt inspection, air content–elevation comparison, borehole and transport temperatures) are thorough. In the main text, the detailed treatment of temperature and air content control (lines 142 ff, including Figures S4–S5 and the

air content–elevation rationale behind Figure 3) is highly technical and could be summarized briefly in the main text, with the full description and the current Figure 3 moved to the Supplement.
We have addressed these notes below in the reviewer's suggested improvement section

D. Statistical treatment and uncertainty handling
The Monte Carlo implementation of the four box model with observational noise and reported standard deviations illustrative, but in my view the fact that only 3 boxes of the 4 box model are constrained by data is a weakness. It is know that in such evaluations models may use the unconstrained boxes in unphysical ways to build a solution. The apparent temporal behaviour of emissions in the tropical south box, including the notable decrease highlighted in the results, may partly reflect the model's structural design and the fact that two tropical boxes are only constrained by one single record with limited temporal resolution and age precision. The robustness of these temporal features should be discussed more critically. Also, I wonder whether other model structural uncertainties (e.g., lifetimes, exchange rates) are fully propagated.
Similar comments have been made by the other two reviewers and we have made substantial changes detailed below

Line 149-156: Uncertainty is discussed here in the main text as follows: "Uncertainties arise in the source strength estimates from observational noise in the ice-core CH_4 concentrations, assumed atmospheric lifetimes, latitudinal box exchange rates, and general structural limitations from the box model's reduced complexity in comparison to the global atmosphere. We have quantified these uncertainties using a Monte Carlo approach that propagates parameter uncertainty and concentration variability as well as sensitivity testing (detailed in methods). While source strength estimates are somewhat sensitive to the assumed oxidation and transport parameters, the enhancement of the tropical southern source strength following the addition of the SCA record is robust to variability from these inputs."

More detail is below regarding specifics in the methods section.

Line 426-450: Uncertainty is now discussed in detail in this section in the methods. We specify the Monte Carlo method here, report each box's average uncertainty in a ± % value and discuss general variability in each box's signal that results from the Monte Carlo method. We have also run sensitivity tests and report the necessary results in here that indicate while the OH loss rate is naturally the largest sensitivity; we use reported values from other similar studies to stay consistent as our goal was to show that the addition of a low latitude core record is important to underestimating historical methane fluctuations. We make the case for the reliability of our results but also note that this box model is simplified relative to the global atmosphere and thus it is expected some limitations will occur.

E. Conclusions
As mentioned above (B and D) I am not convinced that the solution that the authors present is unique, in particular I am concerned by the fact that one box is not constrained by data. The authors do show convincingly how model results can change between having 2 and 3 boxes constrained by data. Therefore I wonder how the results would change if there were also data for the 4th box.
While yes, the improvement of additional data into a model naturally changes the outcomes we are more focused on showing that the previous usage of polar only inversions underestimated tropical sources. We have strived to more clearly show this in the main text (Line 179-181) while noting the model's limitations (Line 149-154) but it's ultimately robust outputs (Line 154-156 and in methods Line 437-450). The record the SCA record is so important is that as noted (Line 53-56 it is the only low latitude record to exist and other cores previously analyzed (Line 40-49) all have notable issues, so there isn't another

historical dataset that would fit within the tropical north box so it must stay interpreted. We also choose to keep the four boxes separate so the air masses remain equal within the model as noted on Line 388. We also chose to constrain the southern box by just capping it at 10 Tg/year as there are previous studies that indicate little to no emissions were occurring and the methane in that latitude is from atmospheric mixing (Line 142-144, 415-417).

Additionally, as mentioned above regarding uncertainty, we have run sensitivity tests detailed in Line 437-450: "Sensitivity tests were conducted on the lifetime and exchange rates across each latitude box by varying each parameter ±30%. While the resulting methane source strength is notably most sensitive to any uncertainty from its own lifetime, the lifetimes are consistent with those used in previous studies[5,53,54]. Transport rates between boxes have modest sensitivity with a maximum ±1.6 Tg/yr between the NH to TN box, ±5.4 Tg/yr between the TN to TS box, and ±17.5 Tg/yr between the TS to SH box (Extended Data Fig. 9).

However, any reasonable perturbation to these transport exchange rates and oxidation lifetimes produces smaller changes than in the relative latitude partitioning of emissions, demonstrating the first-order control of the concentrations as constraints in this box-model. It should be noted that this four-box model is a framework that represents a simplified explanation of atmospheric transport and chemistry (mainly oxidation), and as such, these source strengths are subject to some degree of structural uncertainty that will not be captured by the Monte Carlo approach. Overall, the changes between the two model configurations are more robust in their constraints of CH_4 distribution." An additional figure displaying the sensitivity testing has been added (Extended Data Fig. 9).

F. Suggested improvements

- In the "Constraining Historical CH4 Emissions" section, sharpen the main scientific message: explicitly state what is non trivial about the new constraints (e.g., the magnitude and uncertainty of PI equatorial emissions and their variability) and separate this from the more predictable improvement that comes from having an additional constrained box.

Line 129-135: We have added a description of the model as requested to the beginning of this sentence. The text reads as follows: "To better constrain PI CH_4 latitudinal emission distribution, we applied a four-box atmospheric model with the intention of partitioning sources across each of the four latitude bands. In previous implementations, only polar ice cores have been available and tropical emissions remained implicitly inferred through hemispheric transport assumptions rather than having a direct observational constraint. These polar-only studies highlight the historical dominance of natural emissions in the tropical latitudes[2,5,7], with one such study specifically using polar ice core data and a global box model to estimate tropical emissions[5]."

- Figures and visual presentation

I am a bit disappointed by the Figure quality. This community is know for producing high quality figures that support the narrative, but in this manuscript this is not the case. Suggestions: Merge Figure 1a and 1b into a single multi panel figure. For example, use an inset in 1a to illustrated the 0–1850 CE period, but using a common, clearly labeled x axis and consistent scaling to make temporal comparisons straightforward and reduce redundancy.

Incorporate the current Figure 2 (13C CH4) as an additional panel in the same figure, aligning the timescale with the CH4 panels to visually link concentration and isotope behaviour.

- Move Figure 3 (air content vs elevation) to the Supplement and refer to it briefly in a single main text sentence as supporting evidence that air content is as expected for elevation and shows no sign of modern contamination.
- It would be nice to have a Figure with the location of the SCA site, at least the location needs to be stated precisely. I had to look it up.
Figures 1 and 2 have been merged as suggested by reviewer 2 and reviewer 3. We have re-organized Figure 1 and 2 into a 3 panel figure with a shared x axis and differing y axes to display the changes in the record, with an initial focus on the pre-industrial period, and then a more zoomed out view of the concentrations with the anthropogenic spike highlighted by the core data and the Mauna Loa record. The d13C data is plotted below the concentration data on the same axis. Figure 3 was moved to the extended data (Extended data Fig. 5) and the detail in the text summarized more briefly (Line 125-127). A site figure is now in the supplement for the location of SCA (Extended Data Fig 1) as well as exact coordinates for the drilling site are in summary and in the main text (Line 20, 52).

Isotopic interpretation and wording
- Correct the statement around line 117: −48‰ is the approximate global atmospheric 13C CH4 value, not the weighted global emission value; the latter is more depleted because the sink fractionates strongly, so this distinction should be made explicit.
Line 99-100: Adjusted this sentence to read "A sink driven kinetic isotope fractionation of ~6‰[31] then results in a global atmospheric $\delta^{13}C$-CH_4 value of around -48‰[29].

- Revise the description of the SCA 13C CH4 record (lines 120 ff): the SCA data do not clearly show a pronounced decline into the early 18th century followed by a rapid rise; rather, they are relatively constant through the PI, with the main increase occurring only after ~1850, and the text should be aligned with what is directly visible in the data.
Line 100-104: Due to the small number of samples for the d13C, and the reviewers comment being entirely correct that there is no 'pronounced decline,' the PI trend will not be considered 'constant' as only two data points exist before it starts to fluctuate. We have reworded to read "The overall trend in the SCA $\delta^{13}C$-CH_4 record mirrors that of existing polar records, which have been characterized by depletion through the 1800s followed by a sharp enrichment post 1850 CE (a likely shift to a more fossil fuel based signature[7]) and therefore support that SCA is capturing a global methane signal."

- The reference to "Keeling plots" in the Supplement is misleading because no actual Keeling (or Miller–Tans) plots are shown; clarify what is plotted. If the goal is to infer source signatures or deviations from a background relationship, specify the underlying mathematical model (linear mixing line in concentration–isotope space, intercept definition, regression method), and state explicitly what the SCA points indicate relative to that model. Alternatively, use proper Keeling or Miller–Tans plots with regression lines and uncertainties.
We have removed the plots in the supplement as it does not account for background changes over time and differentiation source changes. The goal of the previous few sentences (Line 95-104) is to discuss the global d13C record with relation to SCA as it indicates a well-mixed global signal. We believe our point is adequately made from lines 95-104 and the addition of this plot did not further our point plus we did not have adequate data to create the Keeling plots properly with SCA. Additionally, we wanted to discuss the low ice availability as requested (Line 108-111), and in so, have removed these sentences to make room for that section.

Placement of technical content
- Move the very detailed technical paragraph beginning around line 142 (borehole temperatures,

transport thermistors, container temperatures, air content curve details) from the main text into the Supplement and leave a concise summary in the main text indicating that temperature control and air content have been verified and support data integrity.
Line 122-127 - The section discussing the temperature/air content/transport etc has been summarized to half of original length and detailed information inserted into the methods section (Line 368-379). Figure 3 has been moved to the supplement and referenced accordingly (now Extended Data . 5).

G. Referencing
The manuscript cites appropriate work on global CH4 budgets, previous low latitude cores, and isotopic and modeling frameworks. Some references are used in multiple contexts without clearly distinguishing when they provide data, methods, or conceptual background; clarifying these roles occasionally would improve readability.
We have clarified the role of key references where they are cited in multiple contexts by adding brief qualifiers (e.g., data, method, framework) at first use or where ambiguity could arise. We have also partitioned the references throughout sentences in a better effort to indicate what is being referenced without adding words given the limit. We applied this selectively to cases where it may have been unclear or where there are a series of references such as the below examples.

Line 30-34: "Atmospheric CH_4 concentrations varied between ~350 and ~800 ppb over the past 800,000 years but have now risen 150% since 1750 CE with 20th-century polar cores recording levels reaching ~1,850 ppb[8]. Most past estimations of CH_4 mixing ratios, fluxes, and emissions still retain uncertainty, highlighting the need for improved constrains on the role of the tropics in CH_4 production and destruction,[5,9-14]."

Line 74-76: "This is particularly apparent in SCA's notable features such as a ~30 ppb increase of CH_4 in late 15th century, followed by a decrease into the 17th century that is also observed in polar ice core CH_4 records[15,20,21,23] (Fig. 1a)."

Line 104-106: "However, the average PI value of $\delta^{13}C$-CH_4 is -49.43±0.14‰, notably ~1‰ more depleted than the PI values recorded in polar cores, whereas after 1850 the $\delta^{13}C$-CH_4 is only ~0.3‰ more depleted than other core records (-48.43±0.14‰)[23,24,32-34]."

Line 125-137: "…the air content of the SCA ice is as expected based on elevation of the drilling site and that of other previously collected cores (Extended Data Fig. 5) [38-42]."

Line 163-168: "Considering current CH_4 production drivers[1,2], past wetland reconstructions[43], the box model results from this study, and previous estimates[5], it is evident that the equatorial regions dominate PI CH_4 production[1,5] but southern tropical sources have been underestimated when only constrained by polar ice core data and that low-latitude core data provides a necessary constraint for resolving previously estimated equatorial CH_4 source strength[43,44]."

The discussion of the atmospheric versus emission weighted 13C CH4 value should also include a specific citation when the corrected interpretation is introduced.
Line 107: Adjusted this sentence to read "A sink driven kinetic isotope fractionation of ~6‰[31] then results in a global atmospheric $\delta^{13}C$-CH_4 value of around -48‰[29]." and the proper reference has been added.

H. Clarity of abstract, introduction, and conclusion

- Scientific argumentation and repetition
Streamline the narrative to avoid restating the same points (e.g., SCA uniqueness, agreement with polar records, and tropical dominance of PI emissions) across abstract, introduction, results, and conclusion; each section should add a distinct layer (motivation, methods, key results, implications) rather than recycling full arguments.
The abstract effectively communicates the existence and importance of the new tropical dataset. The end of the abstract "it confirms the consistent dominance of tropical emissions" is the main scientific result, which is not really unexpected.
Line 13-28: The abstract has been rewritten to a more appropriate general public summary to better introduce both the topic and the studies main conclusions.

Line 24-28: We reworded and clarified why the importance of this core matters, in that is supports the previously thought sustained equatorial historical dominance for CH4 and that the addition of the SCA indicates previous polar core estimates were significantly underestimating the equatorial contribution, establishing low latitude cores as a necessary component for past ch4 variability reconstruction.

Line 30-34: We have additionally, deleted some of the repetitive text from the beginning of the introduction or moved it into the summary paragraph (Line 13-18) to address this comment and the one below.
We have further cut repetitive sentences throughout in an effort to better streamline the narrative as suggested and reduced the overall length of the manuscript by about 200 words.

The introduction is well motivated but somewhat repetitive regarding the problems with previous tropical cores. Condensing this discussion and more sharply highlighting the unique value of the SCA record would strengthen the argumentation. The conclusion could be tightened to focus on a small set of clearly stated, data driven advances: unique tropical record, quantitative increase in inferred PI tropical emissions, improved but still uncertain latitudinal partitioning. and to tone down any over interpretation of model dependent intra box temporal structure
Line 30-46 We have condensed the introduction as noted from the above comment. The conclusion has been edited for repetitiveness (Line 169-183) and to highlight the significant results more appropriately and to make our main point that Huascaran SCA is a unique historical CH_4 record from the tropics and is exceptionally well situated to capture both a global and regional record of CH_4 concentrations and isotope source signature changes as well as its importance into the model because of the continued underestimation of tropical sources when only polar cores are used in past work.

Referee #3 (Remarks to the Author):

This manuscript presents a 2,000-year CH_4 concentration and $\delta^{13}C$-CH_4 record from the Summit Core A (SCA) ice core drilled at Nevado Huascarán, Peru. The authors argue that this record constitutes the first reliable long-term tropical ice core CH_4 record, bridging a known spatial gap between Greenland and Antarctic records. They demonstrate the relationship between the SCA record and specific polar cores during global transitions (anthropogenic and climatic), and show agreement with the Mauna Loa instrumental record. Incorporation of the tropical record into a four-box atmospheric model yields higher inferred tropical methane emissions than estimates based solely on polar data, reinforcing the long-standing hypothesis that low latitude wetlands dominate preindustrial methane variability.

The recovery of a 2000-year tropical ice core CH_4 record is a notable achievement. Previous low-latitude records such as Dasuopu have been compromised by melt, dust-related excess methane, or large

analytical uncertainty, limiting their interpretability. In this context, the SCA record is significant; it provides a direct constraint on tropical methane variability. Where the authors contextualize this study with prior modelling and ice core studies, they may wish to more explicitly delineate how their conclusions extend beyond earlier box-model reconstructions of tropical dominance.

We have added information about how the previous estimates were underestimating the tropical contribution because of the availability of only polar data in various locations through to properly make what is one of our main points (Lines 24-28, 140-144, 157-159, and 176-179).

The analytical methods for CH_4 concentration and isotopic measurements are well established and described (I would suggest some of these detailed, standard methods go in the supplement). The ice core dating, particularly below the annual layer counted portion of the record, needs to be fleshed out. I am not convinced of the dating methods nor accuracy in the deepest part of the record, as very little is shared regarding the oldest part of the developed chronology. I would certainly like to see the glaciochemical data for an annual layer counted section of the record, as well as more information and perhaps a figure (in the supplement) regarding the deepest part of the age scale. The authors do address commonly known complications affecting non-polar ice cores (e.g., dust-driven in situ production, melt layers), and the absence of visible melt layers, low dust and Ca^{2+} concentrations, consistent air content, and agreement with modern atmospheric observations together support the integrity of the record. But again the reliance on CH_4 pattern matching in deeper sections is circular and needs to be addressed.

Line 355-367: We have added detail to the discussion of the development of the timescale in the methods section and have now added to additional supplementary figures (Extended Data Figures 7 and 8) that display the glacio-chemical data for the annual layer counting and 1500 years of d18O matching to the Quelccaya record.

Uncertainties in concentration measurements are reported and propagated through the Monte Carlo simulations of the four-box model. I believe the use of ensemble simulations to account for observational noise and parameter uncertainty is appropriate. However, a clearer discussion of structural uncertainties in the box model itself, particularly the sensitivity of inferred tropical source strengths to the assumed inter-hemispheric exchange rates, the box boundaries, and prescribed OH lifetimes, is warranted.

Similar comments have been made by the other two reviewers. The changes to the text are as follows:

Line 149-156: Uncertainty is discussed here in the main text as follows: "Uncertainties arise in the source strength estimates from observational noise in the ice-core CH_4 concentrations, assumed atmospheric lifetimes, latitudinal box exchange rates, and general structural limitations from the box model's reduced complexity in comparison to the global atmosphere. We have quantified these uncertainties using a Monte Carlo approach that propagates parameter uncertainty and concentration variability as well as sensitivity testing (detailed in methods). While source strength estimates are somewhat sensitive to the assumed oxidation and transport parameters, the enhancement of the tropical southern source strength following the addition of the SCA record is robust to variability from these inputs."

More detail is below regarding specifics in the methods section.

Line 426-450: Uncertainty is now discussed in detail in this section in the methods. We specify the Monte Carlo method here, report each box's average uncertainty in a ± % value and discuss general variability in each box's signal that results from the Monte Carlo method. We have also run sensitivity tests and report

the necessary results in here that indicate while the OH loss rate is naturally the largest sensitivity; we use reported values from other similar studies to stay consistent as our goal was to show that the addition of a low latitude core record is important to underestimating historical methane fluctuations. We make the case for the reliability of our results but also note that this box model is simplified relative to the global atmosphere and thus it is expected some limitations will occur.

The central conclusions (that the SCA record reflects a global atmospheric signal with a strong equatorial imprint, and that tropical emissions dominate preindustrial methane variability) are supported by the data. Some statements could be tempered to distinguish more clearly between well-supported qualitative insights and model-dependent numerical values. Overall I found notable redundancy in the text which could be tightened, with statements being repeated throughout. With a note that modelling is not my own area of expertise, it seems that model sensitivity could be clarified perhaps by a sensitivity analysis in the supplement.

Line 437-442 We had added sensitivity testing as noted above into the methods section which reads "Sensitivity tests were conducted on the lifetime and exchange rates across each latitude box by varying each parameter ±30%. While the resulting methane source strength is notably most sensitive to any uncertainty from its own lifetime, the lifetimes are consistent with those used in previous studies[5,53,54]. Transport rates between boxes have modest sensitivity with a maximum ±1.6 Tg/yr between the NH to TN box, ±5.4 Tg/yr between the TN to TS box, and ±17.5 Tg/yr between the TS to SH box (Extended Data Fig. 9)." Overall, we have restructured the methods between the main text and the detailed section following to tighten our message to the reader and discuss why our data from the model is still consider robust (Line 443-450).

The final note here is that the isotopic dataset is terribly sparse (five samples). While understandable given ice availability, the authors should explicitly discuss how this limits temporal interpretation and whether additional measurements could refine source attribution. It seems clear that this study could be greatly strengthened by more data, and efforts should be made to accommodate this. The figures could use refinement, particularly those combining multiple ice core records (e.g., clearer legends, indication of smoothing or averaging windows).

Line 108-111: We had added a note about the number of samples creating some limitations in the main text.

Line 345-353: There is additional detailed information in the methods that discuss why the ice sampling was so limited for both the concentration data and especially the d13C.

Figures 1 and 2 have been merged as suggested by reviewer 2 and reviewer 3. We have re-organized Figure 1 and 2 into a 3 panel figure with a shared x axis and differing y axes to display the changes in the record, with an initial focus on the pre-industrial period, and then a more zoomed out view of the concentrations with the anthropogenic spike highlighted by the core data and the Mauna Loa record. The d13C data is plotted below the concentration data on the same axis. Figure 3 was moved to the extended data (Extended data Fig. 5) and the detail in the text summarized more briefly (Line 125-127). A site figure is now in the supplement for the location of SCA (Extended Data Fig 1) as well as exact coordinates for the drilling site are in summary and in the main text (Line 20, 52).

Referee #3 (Remarks on code availability):

I downloaded and am currently working to run the code in Python, but want to send my review without further delay. This is not my strong suit so may take me a bit to run successfully.

Formatting Notes

We have made the following formatting edits to align with nature guidelines

- There are now separate Data and Code Availability statements. NOAA data is not able to have a doi at this time (pending approval) and we are prepared to submit to the NSF database upon acceptance and publication, in line with the requirements of the NSF awards used to fund this manuscript's project.
- We have condensed the length of our manuscript in a few places, moved technical details into the methods section, and have re-done the figures so there are 2 multi-panel figures instead of four individual ones in an effort to fit the length requirements better. We have cut approximately 200 words from the main text in an effort to be more concise.
- The summary paragraph has been rewritten for a more general public facing with less repetitive text in the main text introduction. We realize the word count is normally 200, with up to 230 for additional information if needed, our count is at 208 and we would like to not cut anything else if possible.
- The references are split in two with the methods only references following the methods section
- We have added an author contribution section and a no competing interest declaration
- As requested, the supplement figures have been moved into extended data and the figure legends are listed sequentially at the end of the text. As up to ten extended data figures are allowed, we have adjusted accordingly with a pdf containing extended data figures. Extended data figure captions are in the appropriate location in the text as well as in the file Should the manuscript move to publication, we are prepared to submit higher resolution figures as individual jpgs or pdfs if required.
- Figure legends for the main text are listed sequentially after the references in the main text.

We would like to thank all the reviewers for their comments on our manuscript. Line references are for the document with all changes accepted/ tracked changes turned off and responses are in blue italic text.

Referee #1 (Remarks to the Author):
This study's main novelty comes from the tropical ice-core measurements, with the Huascarán SCA record filling a critical gap in historical methane records. The authors have satisfactorily addressed the major concerns raised in my original review. This is particularly evident in the clearer discussion of the uncertainties and limitations of the four-box model, as well as in the addition of sensitivity analyses that explore the effects of methane lifetime and inter-latitudinal exchange rates on the results. They have also appropriately addressed the remaining minor comments, thereby improving the clarity and presentation of the manuscript further. Overall, the revised version is more robust, and I therefore recommend publication.
We thank reviewer 1 for their initial round of comments and second round of review. We note no changes have been requested in this section.

Referee #2 (Remarks to the Author):

The authors have improved the manuscript, especially the figure presentation is much more clear now, and results of a sensitivity study are included, thanks for that. However, the uncertainties are not sufficiently discussed in the main paper, and they may actually question the validity/significance of the inferred emission trends, see below.
We thank reviewer 2 for their comments and suggestions. Details regarding each comment are below with Line numbers and in blue text.

A. Summary of the key results
B. Originality and significance: if not novel, please include reference
The key results are the data from the tropical ice core themselves, and the fact that, according to the argumentation of the authors, they imply larger tropical emissions than inferred without such tropical data.
C. Data & methodology: validity of approach, quality of data, quality of presentation
D. Appropriate use of statistics and treatment of uncertainties
E. Conclusions: robustness, validity, reliability

As mentioned in my first review, upwards revisions in the tropical sources are as such not a strongly surprising result, but it is great that/if novel data that may quantitatively constrain such a revision are now available. With this in mind, it is crucial to discuss uncertainties in inferred emission rates in more detail in the main manuscript. The authors have added a sensitivity study to investigate the uncertainty space (last figure in the extended data), but in the text this is not well discussed. I acknowledge that such an uncertainty investigation is difficult in the absence of many input data, but to me in the present form the discussion of uncertainties is not strong and importantly the uncertainties are not discussed well in light of the key results. One important result from Fig E9 is that the tropical emissions are quite strongly dependent on the assumed tropical lifetimes. In the main paper, the authors conclude that emissions in the tropics need to be adjusted in the tropics, but could a similar change not be achieved with changes in the tropical lifetimes? Or partly emissions and partly lifetimes? This is actually what I understand from Fig E9: If the lifetimes in the tropical boxes were higher, smaller emissions would be needed (which makes sense) and then the inferred increase in emissions that is reported in the main text would be less, potentially far less. Thus, the reported changes in the emissions are only one way to close the budget in the four box model. This questions to some degree the validity, but at least the uncertainty, of the key

scientific results reported in the main text. At the least, this has to be discussed clearly, and if other scenarios can strongly alter the tropical fluxes this has to be included in the uncertainty discussion in the main text.

We have made significant efforts to discuss the model and its resulting uncertainties in both the main text and within the methods section. We have made sure to note the uncertainty of the model in the main text as models are simplified frameworks and not a definitive answer (Line 154-156). We note that the model results are sensitive to change in atmospheric lifetimes, but our chosen lifetimes are backed by previous studies (Line 145-46, 403-405, 445-447). The alteration of the lifetimes in the sensitivity testing do change the specific box being tested, but they do not alter the box's result more than the addition of the SCA record (including the box uncertainty), which shows the previous underestimation from polar only data (Line 147-149, 444-45). We state that this model is a framework and simplified explanation of atmospheric transport (Line 450-453). We have included additional runs as requested and the resulting minor changes that are produced from a run with tropical north input to conclude the model is not sensitive to the interpolation of the TN box (Line 149-155, 399-401, 453-461). More details are discussed further for each specific comment.

The quality of the presentation is improved, but for me there is still way too much repetition, in particular sentences stating that the new data present a contamination free tropical record, or that previous studies did not have tropical data. I think the paper can still be streamlined and shortened quite a lot.

We have made significant efforts to trim repetitive language from this manuscript, with the summary and main text now totaling ~2110 words, where it was previously ~2350. We have especially looked for instances to remove the repetitive statement of the reliability of the record and instead have strengthened the closing sentence to our main text (Line 167-169).

F. Suggested improvements: experiments, data for possible revision

A proper discussion of uncertainties and the robustness of the main results presented is still the main weakness. I would also still suggest that the authors try to investigate in more detail the uncertianties related to having one "empty" box. One possibility would be to provide the concentrations that are returned by the model in the present configuration for the Northern Tropics, and then run the model with synthetic input values that are different from this value, to see how sensitive the model is to the absence of NT measurements. Or add the resutls from the Himalaya glacier??

Reviewer 3 raised similar suggestion about the sensitivity study and uncertainty. We have made the following changes:

Line 149-156: We have now added two additional runs as suggested and discussed in the main text as follows: "Two additional model runs were conducted remove interpolation for the tropical north box using the limited data from the East Rongbuk glacier, and the tropical north data generated from the polar only run as tropical north box inputs These additional runs generate very slight changes (max 3 Tg/yr) in all boxes, indicating the model is not overtly sensitive to the interpolated tropical north box (Extended Data Table 1). We note this model is a simplified framework of atmospheric transport and chemistry, and as such, these source strengths will be subject to some degree of structural uncertainty." The additional runs are further detailed in the methods section on lines 453-461.

We have largely rewritten the section on uncertainty in the main text and in the methods. In the main text the text on lines 139-149 now reads, "Uncertainties in observations and parameters lead to uncertainties in source strength estimates from observational noise in the ice-core CH_4 concentrations, assumed atmospheric lifetimes and latitudinal exchange rates, and general structural limitations from the box model's reduced complexity in comparison to the global atmosphere. We have quantified these

uncertainties using a Monte Carlo approach that propagates parameter uncertainty and concentration variability as well as conducting sensitivity tests (Extended Data Fig. 6; detailed in methods). While source strength estimates are somewhat sensitive to the assumed oxidation and transport parameters, the CH_4 lifetime and exchange rates drawn from previous literature[44,45] show that even a 30% perturbation (drastic for the short CH_4 lifetime) to both the lifetimes and transport exchange rates produces smaller changes than in the relative latitude partitioning of source strengths, demonstrating the first-order control of the concentrations as constraints in this box-model."

We have added a note in the main text that the model is a simplified framework of atmospheric transport to convey that the model results are not absolute but useful (Line 154-156).

We note that we have added discussion on why we used what lifetimes we used (literature backed) and the more comprehensive analysis with the additional model runs has leant a clear picture into the model uncertainty. Further detail for the model uncertainty in the methods section has been almost entirely rewritten for clarity from Lines 433-443.

It may also be useful to add a figure of panel with the average PI concentrations (and uncertainty) and emissions (and better described uncertainty estimate) in addition to the time series, in a way that illustrates the significance of the results in a more intuitive way.
We have displayed the model uncertainties in figure 2as transparent shaded bars around each box's source strength estimate. The uncertainties are now added into extended data Table 1 for each corresponding run. Discussion of changes made in regard to model uncertainty and in text discussion are noted in the above comment in this document.

G. References: appropriate credit to previous work?
Yes

H. Clarity and context: lucidity of abstract/summary, appropriateness of abstract, introduction and conclusions

Title:
Here and elsewhere in the manuscript: "Global and regional" is confusing, remove here and change to tropical elsewhere.
The review makes a valid point with the usage of both of these spatial terms created confusion. We have altered the title to remove 'regional' as the main focus of our paper is the global record from the SCA. We have saved discussion of equatorial contribution to where relevant in the text and have removed all duplicated mentions or made them into separate statements as requested below.

Abstract:
L16: what does "its extent and productivity" refer to?
Line 18: 'its' referred to wetlands, we have updated this sentence accordingly. 'wetland' is now in place of 'its' for clarity.

L21-22: You do not really show this (Amazon basin), it is presented in the text as hypothesis. This sentence can be left out in the abstract
Line 20: This sentence has been removed as requested.

Main text:
L32: why not mention the present atmospheric value instead of the one from polar cores?
Line 31: We have updated the present atmospheric value from the most recently published concentration data from the Mauna Loa station, and the citation is also updated accordingly.

L33-34: reword, and note that you here also mention desctruction, which you do neglect in your analysis (main point)
Line 33: We have removed the word destruction as it is beyond the scope of this paper as we are focused on the record interpretation and the partitioning of latitudinal sources through the pre-industrial period. We do note there is a discussion regarding lifetime sensitivity, but the changing oxidation rates of CH4 over time, its eventual breakdown into CO2 and its competition with other compounds in the atmosphere for OH oxidation is well beyond the scope of our paper.

L48-49: If 15 valid points are available from the Himalaya glacier, how do thye compare to your data. Could they be used to constrain your NT box?
Line 156-160: We have now added two additional runs as suggested with tropical north inputs and discussed in the main text as follows: "Two additional model runs were conducted remove interpolation for the tropical north box using the limited data from the East Rongbuk glacier, and the tropical north data generated from the polar only run as tropical north box inputs These additional runs generate very slight changes (max 3 Tg/yr) in all boxes, indicating the model is not overtly sensitive to the interpolated tropical north box (Extended Data Table 1). We note this model is a simplified framework of atmospheric transport and chemistry, and as such, these source strengths will be subject to some degree of structural uncertainty." The additional runs are further detailed in the methods section on lines 453-461.

Line 46-47: We note that the data from East Rongbuk were mentioned with respect to Greenland (~36 ppb higher). As SCA is 46 ppb higher it sits 10 ppb above the ER data.

L74: global--> tropical
Line 69: We have rewritten this sentence to says 'broadly consistent with polar records' to better fit the intention of the surrounding paragraph.

L77: Why "have the potential"?
Line 72: We have reworded this sentence for clarity from 'have the potential to align' to 'may align' instead. This should allow the discussion of upcoming transitions (i.e. Inca farming etc) without making a sweeping claim that the SCA record has captured these discussed changes.

L83: Unclear sentence: What do you mean by changes?
Line 77-79: We have reworded this sentence for clarity to read as follows: "However, these historical events are not necessarily the direct cause of the observed CH_4 fluctuations but are instead helpful markers to assess concurrently existing records."

L97-8: Can be left out, same and more information in next sentence
Line 88-90: We have condensed these two sentences into one as requested.

L104-6: Looking at Fig 1 is is evident that only the first d13C point is very much lower than from other records, the others are far less than 1per mill lower, taking averages (especially over the strong trend from other locations) is statistically not a good idea! Report the offsets of the individual points relative to the previous records?

Line 93-98: We have rephrased this to discuss each point and comparison datasets instead of averages over time, noting the first point as lower on average than the rest.

L154-6: This is the – in my view biased – summary explanation of the sensitivity study. This part needs to be extended a lot, and the robustness of the signal must be demonstrated better.
Line 142-149: We have rewritten this paragraph to remove any bias statements and better discuss the sensitivity study and also included mention that while the lifetimes are sensitive, they do not change the outcomes more so than the addition of the SCA record and are backed from previous literature as well. The section now reads: "We have quantified these uncertainties using a Monte Carlo approach that propagates parameter uncertainty and concentration variability as well as conducting sensitivity tests (Extended Data Fig. 6; detailed in methods). While source strength estimates are somewhat sensitive to the assumed oxidation and transport parameters, the CH_4 lifetime and exchange rates drawn from previous literature[44,45] show that even a 30% perturbation (drastic for the short CH_4 lifetime) to both the lifetimes and transport exchange rates produces smaller changes than in the relative latitude partitioning of source strengths, demonstrating the first-order control of the concentrations as constraints in this box-model."

L172: "both a global and regional" ◊ tropical
Line 167-169: We have altered the end of this paragraph to align with this suggestion. The final sentence now reads, "The 2,000-year Huascarán SCA record is uniquely positioned to fill a critical gap in historical CH_4 records and improve our understanding of the importance of tropical sources to the global CH_4 distribution."

L175-180: Half of the sentence is repeated twice… remove second sentence.
Line 156-169: Repetitive sentence removed. We have reworked and rewritten much of this entire section (Line 123-169) to be more concise and eliminate repetitive language and noted by this reviewer and reviewer 3.

L335-6: Mention the possible effect of firn fractionation for the deepest and most depleted sample in the main text?
We appreciate the reviewers concern here about firn fractionation, however the implication that the deepest sample is more negative because it is older does not follow general firn fractionation properties. That being said, we acknowledge there are three types of firn fractionation not corrected for in the data. 1) Gravitational: heavier isotopes settle to the bottom of the firn column more rapidly than light isotopes, typically resulting in an enrichment of 0.1 to 0.3 ‰. Correcting the SCA samples for this would make them more negative, slightly increasing the depletion relative to polar records. 2) Thermal: caused during times of rapid temperature change, but typically smaller than 0.1 ‰. 3)Diffusive: lighter isotopes diffusive faster across concentration gradients in the firn column, causing fractionation that is greatest during times of abrupt concentration change. The correction will not account for the relative depletion that would meaningfully alter the SCA data (Buizert et al., 2013).. Apart from the shallowest δ13C-CH4 sample, which we do not interpret in the main text due to age scale uncertainty, the effect is likely much smaller than the negative δ13C-CH4 offset between SCA and other polar records.

For diffusive fractionation we can estimate if we assume barometric pressure of 390 hPa (pressure at 6700 m at -9.5oC), d15N-N2 of 0.2 permil (this gives a diffusive column height of 44m following Buizert et al., 2013), CH4 concentration of 810 ppb (from graph in ref), temperature of -9.5 oC (average from 10

m depth), and a growth rate of 4 ppb/yr (max growth rate from Law Dome at this time is 2 ppb/yr but we are slightly above the Antarctic record). The resulting diffusive fractionation is ~0.58 permil. If we use 2 ppb/yr (from Law Dome) the answer is 0.29 permil. and for 3 ppb/yr is 0.43 permill.

Considering the above we have made the below changes:

Line 96-98: We have adjusted the statement in the main text to read "After considering the potential influence of firn fractionation (see methods)[35] we interpret these $\delta^{13}C$-CH_4 values to indicate a potentially stronger proportion of highly depleted microbial sources during the PI in the equatorial regions."

Line 329-334: We have adjusted the statement surrounding this sampling in the methods section to read "Data are not corrected for gravitational settling[53] or diffusive isotopic fractionation[35] during gas transport in the firn column as the required data do not yet exist. However, we expect these corrections to be small relative to the measured difference in $\delta^{13}C$-CH_4 between SCA and other polar records. We note an expected diffusive fractionation of 0.1 to 0.3 ‰ and where CH_4 growth rates are highest (e.g., the oldest and youngest sample) data may be artificially depleted (between 0.29 -0.43‰ from diffusive fractionation)[35]."

L408: The variables MUST be explained here!
Line 418-422: These variables are now defined in the text as requested.

Fig 1: I would at least note that in panel B the SCA CH4 is LOWER than at NEEM, in contrast to the PI period.
Line 62-63: We have adjusted this sentence to not the SCA falls below the NEEM after the PI period while remaining above the Law Dome record (the only records to persist past the PI cutoff date).

Referee #3 (Remarks to the Author):

I thank the authors for their thorough and constructive response to the initial round of reviews. The revisions have improved the manuscript in several important ways, particularly in terms of clarity, figure presentation, and expanded discussion of model uncertainty and sensitivity. The dataset remains a valuable and technically impressive contribution, providing an important tropical constraint on atmospheric CH_4 variability. Below, I focus on the extent to which the key concerns raised in my initial review have been addressed. *We thank the reviewer for their comments below. We have addressed all requested suggestion and changes.*

Chronology and age model
The authors have made a clear effort to strengthen the description of the chronology, including the addition of new supplementary figures showing glaciochemical data and a section of annual layer counts. This additional material is helpful and represents a meaningful improvement. I remain concerned about the tuning of the deepest portion of the age model; the chronology at depth still relies on alignment to known CH_4 features which is standard. However, those same features are later used to support the interpretation of a globally coherent signal, so there's still a circularity problem here. I encourage the authors to more explicitly distinguish which components of the chronology are independently constrained versus tuned, and to more clearly communicate the uncertainty associated with the oldest part of the record (new extended data fig 8 should indicate uncertainty in this portion of the chronology).
Line 352-374: We have re-computed and re-written the section regarding the SCA ice chronology. The annual layer counting using seasonal variations in the d18O record is still the method used down to 55m.

Beyond 55 meters the d18O record from SCA is matched entirely to the Quelccaya summit ice core (QIC), a well-established record. This was matched with the AnalySeries program and is shown in detail in Extended Data Figure 9. We have chosen to use this approach instead of any matching to CH4 records to remove the issue with circularity and directly note any uncertainty that may arise due to the high vertical compression (Line 356).

Data integrity and glaciochemical context
The authors continue to make a convincing case that the SCA record is not significantly affected by melt, dust-driven in situ CH_4 production, or other common issues in low-latitude ice cores. The addition of supporting figures and expanded methodological detail strengthens this aspect of the manuscript.
Thank you, we appreciate the follow up to the initial comment about the inclusion of geochemical data.

Treatment of uncertainties and model framework
This is an area where the manuscript has improved. The authors have now included a more explicit discussion of uncertainty sources (observational, parametric, and structural) in both the main text and methods, and have added a sensitivity analysis exploring the effects of transport rates and atmospheric lifetimes. The identification of OH lifetime as the dominant sensitivity is useful. I still find that the treatment of structural uncertainty in the four-box model remains somewhat limited. While the sensitivity tests are helpful, they do not fully address concerns about model design. As raised previously (and by other reviewers), this leaves open the possibility that aspects of the inferred latitudinal partitioning may reflect model structure rather than uniquely data-driven constraints. The authors may wish to more explicitly acknowledge this limitation in the main text and to temper interpretation of model-derived temporal variability accordingly.
Reviewer 2 raised similar suggestion about the sensitivity study and uncertainty. We have made the following changes:

Line 149-156: We have now added two additional runs as suggested and discussed in the main text as follows: "Two additional model runs were conducted remove interpolation for the tropical north box using the limited data from the East Rongbuk glacier, and the tropical north data generated from the polar only run as tropical north box inputs These additional runs generate very slight changes (max 3 Tg/yr) in all boxes, indicating the model is not overtly sensitive to the interpolated tropical north box (Extended Data Table 1). We note this model is a simplified framework of atmospheric transport and chemistry, and as such, these source strengths will be subject to some degree of structural uncertainty." The additional runs are further detailed in the methods section on lines 453-461.

We have largely rewritten the section on uncertainty in the main text and in the methods. In the main text the text on lines 139-149 now reads, "Uncertainties in observations and parameters lead to uncertainties in source strength estimates from observational noise in the ice-core CH_4 concentrations, assumed atmospheric lifetimes and latitudinal exchange rates, and general structural limitations from the box model's reduced complexity in comparison to the global atmosphere. We have quantified these uncertainties using a Monte Carlo approach that propagates parameter uncertainty and concentration variability as well as conducting sensitivity tests (Extended Data Fig. 6; detailed in methods). While source strength estimates are somewhat sensitive to the assumed oxidation and transport parameters, the CH_4 lifetime and exchange rates drawn from previous literature[44,45] show that even a 30% perturbation (drastic for the short CH_4 lifetime) to both the lifetimes and transport exchange rates produces smaller changes than in the relative latitude partitioning of source strengths, demonstrating the first-order control of the concentrations as constraints in this box-model."

We have added a note in the main text that the model is a simplified framework of atmospheric transport to convey that the model results are not absolute but useful (Line 154-156).

We note that we have added discussion on why we used what lifetimes we used (literature backed) and the more comprehensive analysis with the additional model runs has leant a clear picture into the model uncertainty. Further detail for the model uncertainty in the methods section has been almost entirely rewritten for clarity from Lines 433-443.

Isotopic constraints
The authors have clarified the interpretation of the $\delta^{13}C$-CH_4 data and corrected several issues in wording. They have also now explicitly acknowledged the limitations imposed by the small number of samples. However, the dataset remains extremely sparse, and this fundamentally limits its interpretive power. While I appreciate the logistical constraints (ice availability), caution in how strongly these data are used to support source attribution is necessary considering these study constraints.
We understand the reviewer's concern with the ice availability for these analysis and interpretation. We discuss the isotopes from Line 94-102, but we have not established it as the main point for our argument of the viability of the SCA record. We note the sample availability, and limited interpretation is discussed on lines 99-102. We also have added a statement about possible firn fractionation into the main text (Line 96-98) to better note issues with sampling alongside the limited sample size.

We addressed firn fractionation according to reviewer 2's comments as noted above.

Figures and presentation
The revisions to the figures are a clear improvement. The consolidation into multi-panel figures, inclusion of uncertainties, and relocation of highly technical material to the Supplement all enhance readability and presentation. There remain sentence fragments, comma misuse, and other minor errors throughout.
We thank the reviewer for their follow up to our previous changes. We have made substantial edits to the entire text to remove minor errors, repetitive language and other grammatical issues.

Conclusions and framing
The manuscript's central conclusions remain largely unchanged: the SCA record supports a strong tropical contribution to atmospheric CH_4 and suggests that previous polar-only approaches underestimated low-latitude emissions. The authors have made efforts to better articulate this point. However, as noted in my initial review, these findings primarily reinforce existing understanding rather than fundamentally revising it. While the new dataset is valuable and important, I encourage the authors to ensure that the strength of their claims remains well aligned with what is directly supported by the data versus what emerges from model-based interpretation.
Line 167-169: We have altered the end of this paragraph to align with this suggestion from reviewer 2 and reviewer 3. The final sentence now reads, "The 2,000-year Huascarán SCA record is uniquely positioned to fill a critical gap in historical CH_4 records and improve our understanding of the importance of tropical sources to the global CH_4 distribution."

Overall assessment
The manuscript has improved following revision and presents an important and unique dataset. Several of the concerns raised in the initial review - particularly regarding clarity, uncertainty discussion, and presentation - have been addressed. Some key issues remain only partially resolved, most notably the robustness and independence of the deepest chronology, the sample constraints, and the structural

limitations of the four-box model and their implications for interpretation. These do not invalidate the study, but they do warrant continued caution in interpretation and clear acknowledgment of limitations.
We thank the reviewer for their continued suggestions for improving our manuscript and note our changes above to address their comments and strengthen the overall narrative of this study.

<u>General editorial guidelines:</u>

STATISTICS: Authors should ensure that any statistical analysis used is sound and that it conforms to the journal's guidelines (see https://www.nature.com/nature/for-authors/formatting-guide for guidance).
We have ensured this manuscript meets the statistical guidelines set for this paper including the n value of the data (Line 304), the measure of variability (Line 313-314) and whether the variability is sem or sd (Line 314). All data generated from this study has been reported fully and made available through a public repository.

FIGURE LEGENDS: These should be listed sequentially after the references in the main text and not in the figures files. Each legend should begin with a brief title for the whole figure and continue with a short description of each panel and the symbols used. Any error bars in the figures must be defined (for example, s.d., s.e.m.) and the value of n indicated; see https://www.nature.com/nature/for-authors/formatting-guide for further explanation.
We have placed the figure legends in the appropriate location and defined all error bars in the captions are required.

DISPLAY ITEMS: We ask that you take stock of all the data that have been generated throughout the review process and ensure that only the data most central to the conclusions are presented in the main figures. Figures should be comprehensible to readers in other or related disciplines, and assist their understanding of the paper. We encourage authors who are describing complex processes to include a schematic of the main finding as part of the Extended Data to aid readers unfamiliar with the immediate discipline. Figures should be as small and simple as is compatible with clarity. All panels of a figure should be logically connected; each panel of a multipart figure should be sized so that the whole figure can be reduced by the same amount and reproduced on the printed page at the smallest size at which essential details are visible. For guidance, Nature's standard figure sizes are 89 mm (one column), 120 mm (one and a half columns), or exceptionally 183 mm (two columns) wide; the full depth of a Nature page is 247 mm. All panels of figures should be presented on a single page and assembled into a rectangular shape for publication; please indicate any essential alignments (parts horizontal, vertical, spacings of stereo pairs, etc.). Tables should be prepared using the Table menu in Microsoft Word.
In the pursuit of satisfying reviewer comments and ensuring clarity, we have generated a new Ex Data Fig for the ice chronology and replaced the initial Ex Data Table 1 with information about the model variability. The initial Ex Data Table 1 variables are now defined in the methods section as requested by a reviewer comment. All figures have been generated to standardized sizes.

FIGURE FORMATTING: Lettering in all figures (labelling of axes and so on) should be in uniform, sans-serif font, in lower-case type, and large enough to permit substantial reduction for publication (minimum font size 5 pt). Separate parts of a figure are labelled a, b, etc. Units have a single space between the number and the unit, and follow SI nomenclature or the nomenclature common to a particular field. Thousands are separated by commas (1,000). Unusual units or abbreviations are defined in the legend. Scale bars rather than magnification factors should be used.

All figures with separate parts have been labeled a,b, etc in a sans-serif font that is larger than 5pt. All units have been labeled, and thousands are separated by commas. Any abbreviations are defined in the legend as necessary.

IMAGE PRESENTATION: Authors should be aware that any image provided for publication, either in print or online (as Extended Data or Supplemental Information), may be subject to a quality control process to check for image integrity and manipulation. For a full discussion of our standards regarding how images should be prepared and presented, see www.nature.com/authors/editorial_policies/image.html.
We have prepared all figures under the expectation to meet the Nature requirements for quality control and checking. All figures have adjoining csv files for plotting the data.

EXTENDED DATA: Please remove the legends from your Extended Data file.
All legends have been removed and put in the main text document.

SUPPLEMENTARY INFORMATION: Supplementary Information is online-only material published with the manuscript (https://www.nature.com/nature/for-authors/supp-info). For most papers, there should be no need for Supplementary Information beyond that already provided as Methods and Extended Data, the aim being to avoid unnecessary fragmentation of the paper online. Exceptions to this rule include large datasets that cannot be accommodated within Extended Data; video material; and more complex "Supplemental Methods" (and any associated references) that do not readily fit within the constraints of the Methods/Extended Data formats.
Please note that after the paper has been formally accepted you can only provide amended Supplementary Information files for critical changes to the scientific content, not for style. You should clearly explain what changes have been made if you do resupply any such files. *We do not have supplemental material for this paper, all extra data is collated into the extended data section as a figure or table.*

SOURCE DATA: To further increase transparency, we encourage authors to provide, in spreadsheet form, the data underlying the graphical representations used in the figures. This is in addition to our well-established data-deposition policy for specific types of experiments and large datasets. Readers of the online manuscript will be able to access the Source Data directly from the figure legend. Spreadsheets can only be submitted in .xls, .xlsx or .csv formats. One file per figure is permitted; thus, if there is a multi-panelled figure the Source Data for each panel should be clearly labeled in the csv/Excel file; alternatively the data for a figure can be included in multiple, clearly labeled sheets within an Excel file. File sizes of up to 30 MB are permitted; however, it is expected that the vast majority of Source Data files will be considerably smaller than this. When submitting these files with your manuscript, please select the file type "Source Data" and use the title field in the file description tab to indicate the figure to which the Source Data pertain. *All source data for the figures and extended data figures have been submitted with the manuscript as requested and are available in the noted Zenodo repository.*

Referees' comments:

Referee #2 (Remarks to the Author):

Thanks for considering the suggestions in preparing a new version of the manuscript. The scientific robustness of the manuscript has strongly improved, in particular the description of uncertainties and framing of the results is now adequate in my view.
We thank the reviewer for their comments to better improve our manuscript. Our responses are below in blue text with the line numbers corresponding to the final document.

I find the language and sentence constructions sometimes still hard to follow. Some sentences are incomplete, (too) long, sloppy or grammatically wrong. Nominal writing style could be exchanged to verbal style at several places to improve readability. Few examples for clarifying language are included below, but there are more language issues that should be improved. Please review carefully among the authors.
We have taken another pass through the manuscript, both the main text and methods and have rewritten, rephrased, and re organized a great deal of the text to ensure clarity and eliminate run on sentences. The most substantive rewrites are in the second to last paragraph of the main text and the methods section discussing the box model.

Check the use of "equatorial" versus "tropical". For the site, it may be adequate to refer to it as equatorial, but for sources tropical may be the better term.
We thank the reviewer for this comment. We note that previously there was discussion about using equatorial instead of tropical. We have adjusted the main text appropriately to use equatorial at Lines 24, 25, 70, and 173 to discuss location as suggested. We use these mostly when discussing unknowns of the exact type of sources the CH4 is from. We use tropical when discussing methane's general dominance as a wetland produced emission. In the box model we have chosen to leave a tropical as it refers to the location of the two tropically located boxes.

It is hard to find in which parameters are actually varied in the 1000 members of the Monte Carlo simulations. The main paper mentions "parameter uncertainty and concentration variability". Line 831 mentions 5 ppb concentration uncertainty. Lines 825/26 mention life times and exchange times, are these also varied, and over which range? If not, which other parameters are varied? It would be useful to summarize this clearly at some place.
Line 412-415 We have added the following summary sentence for clarification: *"Within the Monte Carlo framework, the observational inputs (CH_4 concentrations) are varied across iterations, the atmospheric lifetimes and transport exchange rates are held, with their influenced assessed through the sensitivity analysis described below."*

Spoecific comments (line numbers refer to "track changes" document on server)
1) Line 30-31: Thanks for including the current atmospheric values, but is this correct? Please have a look at https://gml.noaa.gov/ccgg/trends_ch4/. We are not at 1964 ppb yet as global average, and the 2024 value seems to be about 1925 ppb. From the title of the cited reference it sounds that the value derived there is for the NH (only NH stations are mentioned). I suggest using the global average value in this introduction. Also, the 800 ppb maximum cited for the past 800,000 years is not adequate as value for year 1750 when assessing the increase from 1750-present. Meinshausen et al (Table 6) state 731 ppb for year 1750. Using the more adequate numbers the increase since 1750 is > 160%.
Line 31 – We have updated according to the NOAA database with the global average for 2025, which is 1,935 ppb. Considering the 731 ppb value the percent increase has been shifted to ~160%.

2) Line 89: consider a different word than "reference", maybe simply "record". Reference has often more

specific meaning in science. Also, in the description of Fig 1, I miss the most obvious feature that SCA is higher than the polar cores in the PI period. It only comes in line 98, consider moving this up?
Line 56 – We have changed reference to record as suggested.
We have chosen to leave the discussion of the SCA record where it is in main text as moving up in this section would impede the discussion of the polar cores. It is mentioned as soon as the SCA record is brought up on Line 69.

3) Line 97: just by eye, the NEEM-SCA difference seems to be much larger than 20 ppb, at least in the latter part. Make sure you specify the period and check the value, in order not to confuse readers (it is confusing to me).
Line 71-72: This ~20 ppb difference is noted for the period past the Pre Industrial (post 1850), where the SCA record shifts below the NEEM record. We have double checked and adjusted our average to 30 ppb using the same temporal overlap for comparison.

4) Line 119: please find a better construction than "global fluctuation similarity"
Line 90: We have reworded this phrase to "the SCA record reflects global fluctuations"

5) Line 259: Difficult to follow the argumentation: I assume you want to say that this demonstrates that the observed changes in concentrations must be to first order driven by source changes.
Line 153-156: We have adjusted and shortened this for clarity:
"Source strength estimates are somewhat sensitive to the assumed oxidation and transport parameters, but even a 30% perturbation in CH_4 lifetime and exchange rates drawn from previous literature[44,45] produces smaller changes than in the relative latitude partitioning of source strengths from the inclusion of the SCA data."

6) Line 260: Incomplete sentence. Conducted to remove? The entire sentence could be clarified
Line 156-161: We have added the missing period on line 156. The sentence has been rephrased to the following: "*To investigate the sensitivity of the tropical north box interpolation, two additional model runs were conducted using the limited data from the East Rongbuk glacier and the tropical north data generated from the polar only run as tropical north box inputs. These additional runs generated very slight changes (max 3 Tg/yr) in all boxes, indicating the model is not overtly sensitive to the initial interpolated tropical north box (Extended Data Table 1)."*

7) Line 265: …the derived source strengths are …
Line 162: We have changed the phrasing from 'source strengths' to 'derived source strengths'

8) Line 268: …. contributions from polar records only.
Line 165: We have changed this sentence to '*Previous studies have only estimated historical tropical contributions from polar records*[5,7]*."*

9) Line 276:…. from equatorial CH4 sources.
Line 173: We have added the word 'sources' after 'equatorial CH4'

10) Final sentence: This sounds to me like an opening sentence describing the potential. I suggest to modify this final sentence to: The 2,000-year Huascaran SCA record fills a critical gap in historical CH4 records and improves our understanding of the importance of tropical sources to the global CH4 distribution.
Line 174-176: We have reworded as suggested

11) Line 490: Core
Line 287: Fixed type to 'core'

12) Line 511: Is the bath refrigerated? Please reword.
Line 309-312: We are unsure how to add any more specifics into this sentence. All instances of the bath being mentioned in this section note the state of the water. Line 309 and 312 are ‘refrigerated bath (-60C)’ and Line 310 says ‘warm water bath (50C).

13) Line 514: remove “in”
Line 312: Removed ‘in’

14) Line 569: Why did you remove the per mill sign?
Line 343: Added the per mill sign

15) Line 630: Do you mean little ice age?
Line 380: This section in the methods discusses changes in the CH4 record that were used to help construct the ice core timescale in comparison with the Quelccaya Ice Cap d18O record. The words ‘ice age’ on Line 374 are in reference to the ice age versus gas age in the core as the CH_4 record timescale is offset from the ice timescale due to the fact that air is trapped at depth in the firn. We discuss s CH4 fluctuations with respect to the Little Ice Age in the main text on lines 82-83.

16) Line 654: Can you specify the “defined transport fluxes”? I suggest to move the information from Line 825/26 up to line 654.
Line 398-402: We have moved up the defined lifetimes and fluxes to the beginning of this paragraph as suggested.

17) Line 659: I would say the 16 g/mol for molar mass of methane is not an “assumption”
Line 406-407: A good point. We have added the word use so the sentence now reads: “. *For all model versions we assume a total tropospheric mass of 1.78 x*10^{20} *moles of dry air and use a* CH_4 *molar mass of 16 g/mol.*

18) Line 911: Backed by
Line 460: This sentence was rewritten for clarity with regard to a previous comment above and now reads *“This places our ±30% range within the uncertainty found in full chemical transport models. Transport rates between boxes, are grounded in previous literature44,45.”*

19) Line 919: in the with ??
Line 465: Changed to ‘in the run with’

20) Line 921: “as well as less in the other boxes” – rewrite
Line 461 – We have rephrased this to ‘with a max of 3 Tg/yr.’

21) Line 922: Unclear sentence, …with polar only to the inclusion of the SCA data -- ??, also the last addition, please rewrite and read carefully!!
Line 462-464 We have rephrased this sentence to: “*Overall, the changes between the polar only model configuration and the inclusion of the SCA data are more robust in their constraints of* CH_4 *distribution that they are sensitive to the interpolation of the TN data.”*

Referee #3 (Remarks to the Author):

I thank the authors for their careful and thorough response to my second-round comments. The revisions represent a meaningful improvement to the manuscript across several dimensions. The chronology section has been substantially reworked. The decision to anchor the deep portion of the age model to the

Quelccaya summit ice core rather than CH_4 features directly addresses my concern about circularity, and the additional Extended Data figure showing this alignment is a strong addition. The uncertainty associated with the deepest portion of the record is now more clearly communicated. The expanded uncertainty treatment is appreciated. The authors have appropriately rewritten the uncertainty discussion in both the main text and methods, now explicitly identifying observational, parametric, and structural sources of uncertainty. The addition of the two supplementary model runs effectively demonstrates that the model is not overtly sensitive to the interpolated tropical north box, addressing a concern raised across multiple rounds of review. The Monte Carlo propagation approach and the demonstration that even a 30% perturbation to lifetimes and transport rates produces changes smaller than the effect of adding the SCA record together provide a reasonable case for the robustness of the main findings. I note that structural uncertainty in the four-box model remains an inherent limitation of this study, and some aspects of the inferred latitudinal partitioning may still reflect model design. However, the authors have now acknowledged this explicitly and have appropriately tempered their conclusions. The revised closing sentence accurately reflects what the data directly support. The isotopic data remain sparse, and the authors have acknowledged this limitation along with the potential influence of firn fractionation. The interpretation I would say is now suitably cautious. The figures, presentation, and editorial formatting all meet the required standards. I am satisfied that the authors have addressed my concerns to the extent feasible given the available data, and I recommend acceptance for publication. Alison Criscitiello

We would like to thank this reviewer for their comments and careful review of our subsequent changes. The notes about the timescale and model uncertainty in particular have helped greatly in our improvement of this manuscript.
